# NLRX1 is a key regulator of immune signaling during invasive pulmonary aspergillosis

**DOI:** 10.1371/journal.ppat.1008854

**Published:** 2020-09-21

**Authors:** Bridget Kastelberg, Nuria Tubau-Juni, Tariq Ayubi, Austin Leung, Andrew Leber, Raquel Hontecillas, Josep Bassaganya-Riera, Shiv D. Kale

**Affiliations:** Nutritional Immunology and Molecular Medicine Institute, Blacksburg, Virginia, United States of America; Rutgers New Jersey Medical School, UNITED STATES

## Abstract

*Aspergillus fumigatus* is an opportunistic fungal pathogen of immunocompromised patient populations. Mortality is thought to be context-specific and occurs via both enhanced fungal growth and immunopathogenesis. NLRX1 is a negative regulator of immune signaling and metabolic pathways implicated in host responses to microbes, cancers, and autoimmune diseases. Our study indicates loss of *Nlrx1* results in enhanced fungal burden, pulmonary inflammation, immune cell recruitment, and mortality across immuno-suppressed and immuno-competent models of IPA using two clinically derived isolates (AF293, CEA10). We observed that the heightened mortality is due to enhanced recruitment of CD103+ dendritic cells (DCs) that produce elevated amounts of IL-4 resulting in a detrimental Th2-mediated immune response. Adoptive transfer of *Nlrx1-/-* CD103+ DCs in neutropenic NRG mice results in enhanced mortality that can be ablated using IL-4 neutralizing antibodies. *In vitro* analysis of CD103+ DCs indicates loss of Nlrx1 results in enhanced IL-4 production via elevated activation of the JNK/JunB pathways. Interestingly, loss of Nlrx1 also results in enhanced recruitment of monocytes and neutrophils. Chimeras of irradiated *Nlrx1-/-* mice reconstituted with wild type bone marrow have enhanced neutrophil recruitment and survival during models of IPA. This enhanced immune cell recruitment in the absence of Nlrx1 is mediated by excessive production of CXCL8/IL-8 family of chemokines and IL-6 via early and enhanced activation of P38 in response to *A*. *fumigatus* conidia as shown in BEAS-2B airway epithelial cells. In summary, our results point strongly towards the cell-specific and contextual function of Nlrx1 during invasive pulmonary aspergillosis and may lead to novel therapeutics to reduce Th2 responses by CD103+ DCs or heightened recruitment of neutrophils.

## Author summary

Fungal infections are mitigated and controlled in part by a robust immune response and generation of reactive oxygen species. In certain instances, the immune response may become harmful to the host. Nlrx1 is a known negative regulator of inflammatory aspects of the immune system in response to viruses, bacteria, and cancers. In this study we describe the novel importance of Nlrx1 in controlling and fighting fungal infections in two different host cell populations through two distinct mechanisms. Nlrx1 may function as a future target to mitigate inflammation and immunopathogenesis during fungal pulmonary infection as well as enhance beneficial neutrophil recruitment.

## Introduction

Invasive pulmonary aspergillosis (IPA) is an aggressive infection of the respiratory system predominantly mediated by the saprophytic fungus *Aspergillus fumigatus*. Individuals who are most at risk include immune-suppressed patients, and those with limited production of reactive oxygen species [1–4]. Disease manifestation is thought to be mediated largely by the type of immune suppression. Conversely, there is a growing appreciation for the role of immunopathogenesis during *A*. *fumigatus* colonization [5, 6]. Current therapeutic strategies involve the targeting of ergosterol biosynthesis by azoles, β-glucans synthesis by echinocandin, and membrane leakage via polyenes such as amphotericin B [4, 7, 8]. Novel therapeutic strategies are urgently needed due to the growing incidences of anti-fungal resistance amongst clinical and environmental isolates [9–11]. High degree of phenotypic heterogeneity in context to growth and stimulation of host immunity has also been observed amongst *A*. *fumigatus* isolates [12–14]. Identifying therapeutic targets that have efficacy in killing disparate isolates is an important hurdle to address. Here we present Nlrx1 as a prospective host-targeted therapeutic for fungal infections that is involved in regulating the intensity and type of immune response to *A*. *fumigatus* in several pre-clinical models of IPA.

Nlrx1 (NOD9) is a critical regulator of immune signaling, metabolism, autophagy, and cell death in response to viruses, bacteria, eukaryotes, cancer, autoimmunity, and tumors [15–23]. Nlrx1 functions as a negative regulator of innate immunity towards a number of DNA viruses and HIV-1 through endogenous binding to the DNA-Sensing adaptor STING. Binding prevents TANK-binding kinase (TBK1) activation via STING in response viral DNA. This function ultimately results in a loss of beneficial IFN-1 induction [24]. Likewise, Nlrx1 also antagonizes NF-κB mediated inflammatory responses via binding TRAF6 and/or the IKK complex [18,19]. Nlrx1 has been shown to directly bind to a number of pathogen and danger associated molecular patterns via its C-terminal three helix bundle resulting in enhanced immune signaling and inflammation [25, 26]. Furthermore, Nlrx1 plays an important role in the killing of *Histoplasma capsulatum* through LC3-associated phagocytosis (LAP) via TUFM/ATG5-ATG12 interactions [23]. A N-terminus targeting sequence has the ability to translocate NLRX1 to the mitochondria where it has been shown to also interact with ubiquinol-cytochrome *c* reductase core protein II (UQCRC2), a component of mitochondrial complex III [27]. The multiple mechanistic functionalities of Nlrx1 suggests it is a critical immuno-metabolic hub in controlling both immune signaling in response to pathogen and cellular dangers as well as reactive oxygen species production. Based on these findings and our prior RNA-Seq analysis [28], we became interested in Nlrx1 as a regulator of immune signaling in response to fungal infections, specifically invasive pulmonary aspergillosis.

## Results

### Loss of *Nlrx1* results in elevated fungal burden across 4 mouse models of pulmonary fungal infection

**Fig 1:**
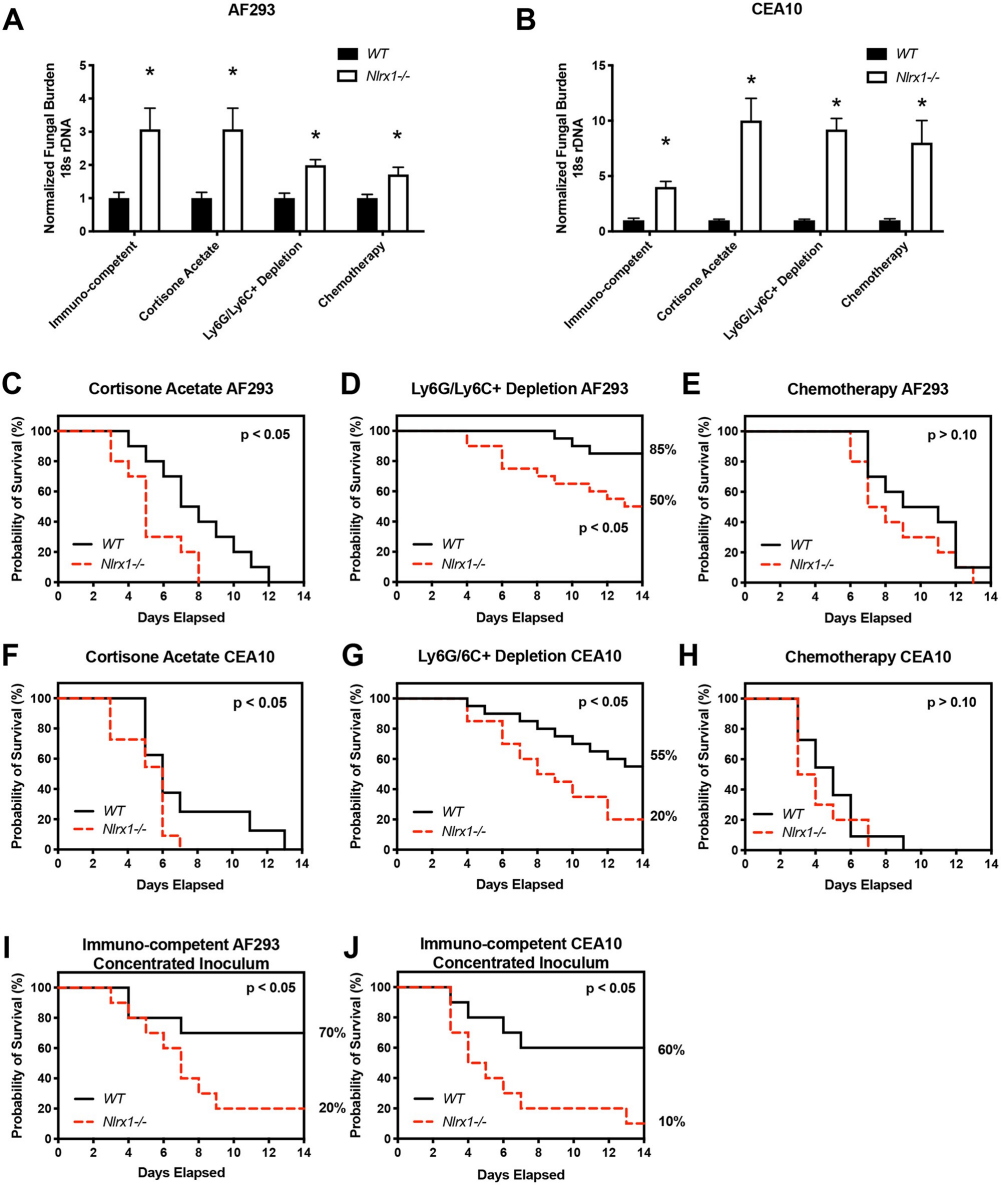
Measurement of fungal burden and survival during mouse models of invasive pulmonary aspergillosis. Freshly harvested conidia from either the AF293 or CEA10 isolate were delivered via aerosolization (12 mL, 1 X 10^9^ conidia/mL) to wild type and *Nlrx1-/-* mice under immuno-competent or immuno-suppressive conditions including antibody based induction of neutropenia (Ly6G/Ly6C+ depletion), cortisone acetate treatment, and chemically induced leukopenia (Chemotherapy). Normalized fungal burden from mice that were humanely euthanized on day 3 post inoculation with either the (A) AF293 isolate or (B) CEA10 isolate. Kaplan-Meier survival curve of wild type and *Nlrx1-/-* mice aerosol inoculated with either the (CDEI) AF293 or (FGHJ) CEA10 isolate of *A*. *fumigatus* in various immune suppressive states. Kaplan-Meier survival curve of wild type and *Nlrx1-/-* mice inoculated with concentrated (I) AF293 or (J) CEA10 conidia (1 X 10^9^ conidia/ 25μL) via non-invasive intra-tracheal instillation. (AB) Asterisk denotes statistical significance, *P* < 0.05 Mann-Whitney U test. Error bars indicate standard deviation. N = 8–10 per experimental group. (C-J) Statistical significance was determined using the log-rank (Mantel-Cox) test. N = 10–20 per experimental group.

To determine the physiological relevance of *Nlrx1* during pulmonary fungal challenge we aerosol inoculated immuno-competent and immuno-suppressed wild type and *Nlrx1-/-* mice (C57BL/6) with the AF293 or CEA10 isolate of *A*. *fumigatus* (1 X 10^9^ conidia/mL, 12mL/ ~1hr). Mice were immuno-suppressed with either cyclophosphamide and cortisone acetate treatment (Chemotherapy), antibody-based depletion of Ly6G/Ly6C+ cell populations (predominantly neutrophils) using the RB6-8C5 antibody (Ly6G/Ly6C+ depletion), or cortisone acetate treatment. Fungal burden was assessed through measurement of 16s rDNA by qPCR (AF293) or digital droplet PCR (ddPCR, CEA10) on day 3 post-inoculation. A 2–3 fold increase in normalized fungal burden was observed for *Nlrx1-/-* mice in comparison to wild type for all four models using the AF293 isolate (*P* < 0.05, Mann Whitney U test (MWUT), Fig 1A). A 5–10 fold increase in normalized fungal burden was also observed for *Nlrx1-/-* mice in comparison to wild type for the CEA10 isolate for all four models (*P* < 0.05, MWUT, Fig 1B). The loss of host *Nlrx1* results in a significant increase in pulmonary fungal burden in comparison to wild type regardless of immune status for two distinct clinical isolates of *A*. *fumigatus*.

### *Nlrx1*-/- mice have reduced survival in specific models of IPA

We then utilized these 4 models of *A*. *fumigatus* aerosol challenge to assess if *Nlrx1* affected survival of mice over a 14-day period. We noted a significant increase in mortality for *Nlrx1-/-* mice using the steroid treatment and antibody induced Ly6G/Ly6C+ depletion models when challenged with either the CEA10 or AF293 isolate (*P* < 0.05, Log-rank (Mantel-Cox) test, Fig 1C, 1D, 1F and 1G). We did not observe any statistically significant differences in rate of mortality between the wild type and *Nlrx1-/-* mice when using the chemotherapy models of IPA for either isolate (*P* > 0.10, Log-rank (Mantel-Cox) test, Fig 1E and 1H). Mock aerosol-inoculated, immuno-suppressed mice of either genotype did not succumb to mortality indicating enhanced mortality was not due simply to immune suppression (S1A Fig). Aerosol inoculation of immuno-competent wild type and *Nlrx1-/-* mice also resulted in an absence of mortality (S1BC Fig), while delivery of a concentrated inoculum via non-invasive intra-tracheal instillation (1 X 10^9^ conidia/ 25 μL) of the CEA10 or AF293 isolate resulted in enhanced mortality for *Nlrx1-/-* mice in comparison to wild type (*P* < 0.05, Log-rank (Mantel-Cox) test, Fig 1I and 1J). Based on these results we conclude loss of *Nlrx1* increases mortality in the steroid and neutropenia models of IPA for both isolates. The effects of *Nlrx1* on mortality appear to be negated in the chemotherapy model implying relevant cell populations or functions may be depleted or marginalized when cyclophosphamide is administered. The importance of *Nlrx1* in context to mortality is also dependent on the concentration of inoculum as a highly concentrated dose of either isolate resulted in enhanced mortality in the *Nlrx1-/-* background.

### Loss of *Nlrx1* results in enhanced pulmonary inflammation and infiltration

**Fig 2:**
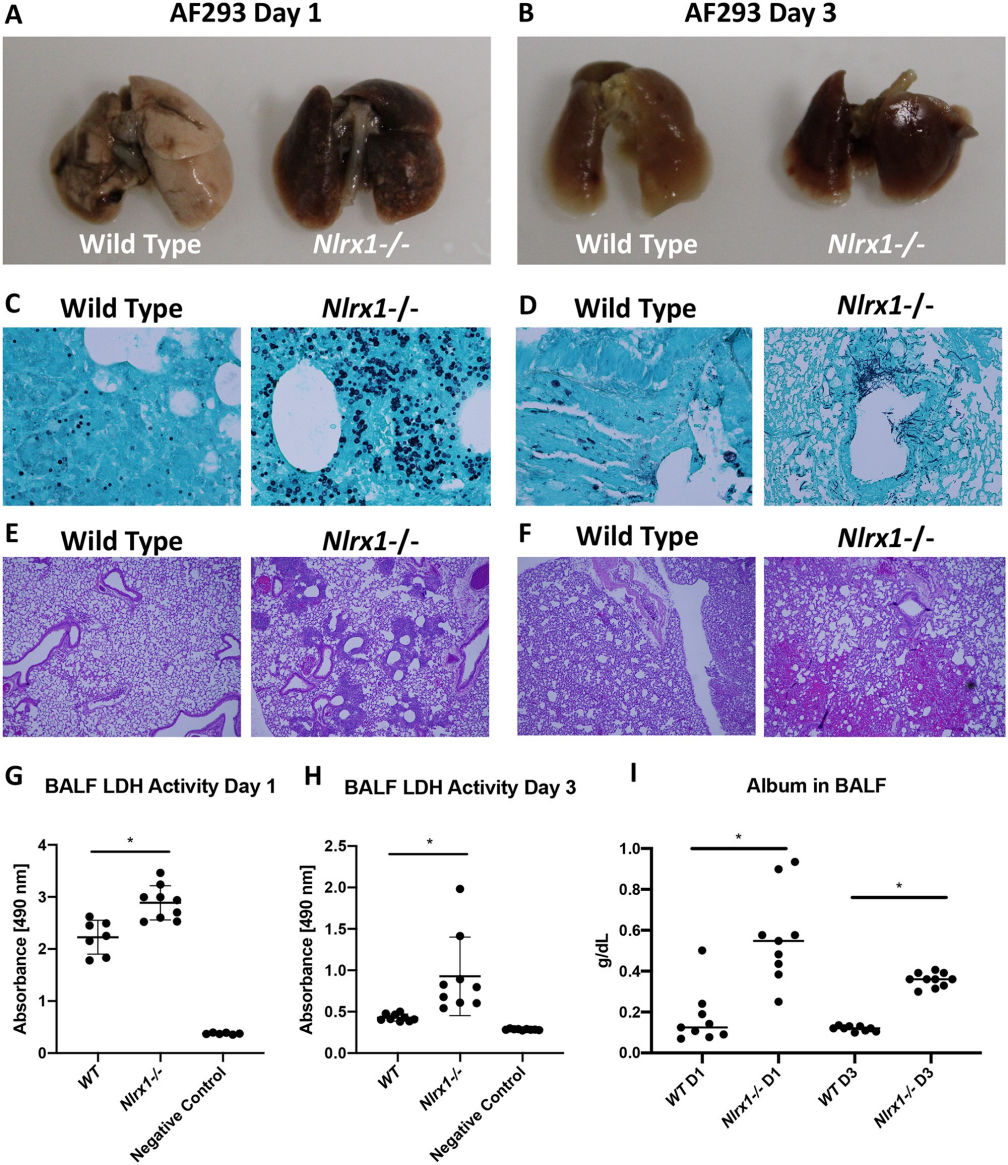
Characterization of pulmonary inflammation and fungal burden on day 1 and 3 post inoculation with AF293. Freshly harvested conidia from the AF293 isolate were delivered via aerosolization (12 mL, 1 X 10^9^ conidia/mL) to immuno-competent wild type and *Nlrx1-/-* mice. (AB) Gross anatomy of excised lungs perfused with formalin on day 1 and 3 post inoculation. (CD) Grocott’s methenamine silver stain and (EF) hematoxylin and eosin stain of lung sections from wild type and *Nlrx1-/-* mice on day 1 and 3 post inoculation. (GH) Lactate dehydrogenase activity in bronchoalveolar lavage fluid (BALF) from wild type and *Nlrx1-/-* mice on day 1 and 3 post inoculation. (I) Albumin levels in BALF from wild type and *Nlrx1-/-* mice on day 1 and 3 post inoculation. Asterisk denotes statistical significance, *P* < 0.05, Mann-Whitney U test. Error bars indicate standard deviation. N = 6–10.

The enhanced fungal burden and increase in model specific mortality observed for *Nlrx1-/-* mice suggested a deficiency in the killing and clearance of fungal hyphae and conidia as well as a possible role for enhanced immunopathogenesis in disease and mortality. We examined lungs from immuno-competent mice on days 1 and 3 post aerosol-inoculation (Fig 2A and 2B). Visual inspection of inoculated lungs on days 1 and 3 was startling as wild type lungs contained patches of localized inflammation and necrosis, while *Nlrx1-/-* lungs looked blackened and necrotic throughout (Fig 2A and 2B). Analysis of fungal load in *Nlrx1-/-* mice by GMS staining suggested enhanced conidial swelling and germination in comparison to wild type on day 1 (Fig 2C). Hematoxylin and eosin (H&E) staining indicated mild and focal PMN infiltration and congestion in wild type mice on day 1 post treatment (Fig 2E). Rampant necrosis of the airway epithelium, severe infiltration of PMNs around airways, and severe congestion and presence of RBCs in alveolar spaces was observed on day 1 in *Nlrx1-/-* mice (Fig 2E). By day 3, elongating hyphae could be observed in the primary airways and occasionally in alveoli for *Nlrx1-/-* mice (Fig 2D). In contrast, nearly all fungal load was cleared in immuno-competent wild type mice and only in a few instances were un-swollen conidia observed (Fig 2D). H&E staining of wild type lungs on day 3 post challenge indicated moderate infiltration of PMNs, enhanced congestion and the presence of RBCs in alveolar spaces (Fig 2F). Enhanced infiltration of PMNs, severe congestion, and edema was noted throughout the tissue as well as necrosis throughout primary and secondary airways for *Nrx1-/-* mice (Fig 2F). Both lactate dehydrogenase (LDH) activity and albumin levels in bronchoalveolar lavage fluid were significantly elevated in *Nlrx1-/-* mice on day 1 and day 3 post inoculation with the AF293 isolate, further providing a quantification of enhanced cell death and apoptotic responses (*P* < 0.05, MWUT, Fig 2G, 2H and 2I).

**Fig 3:**
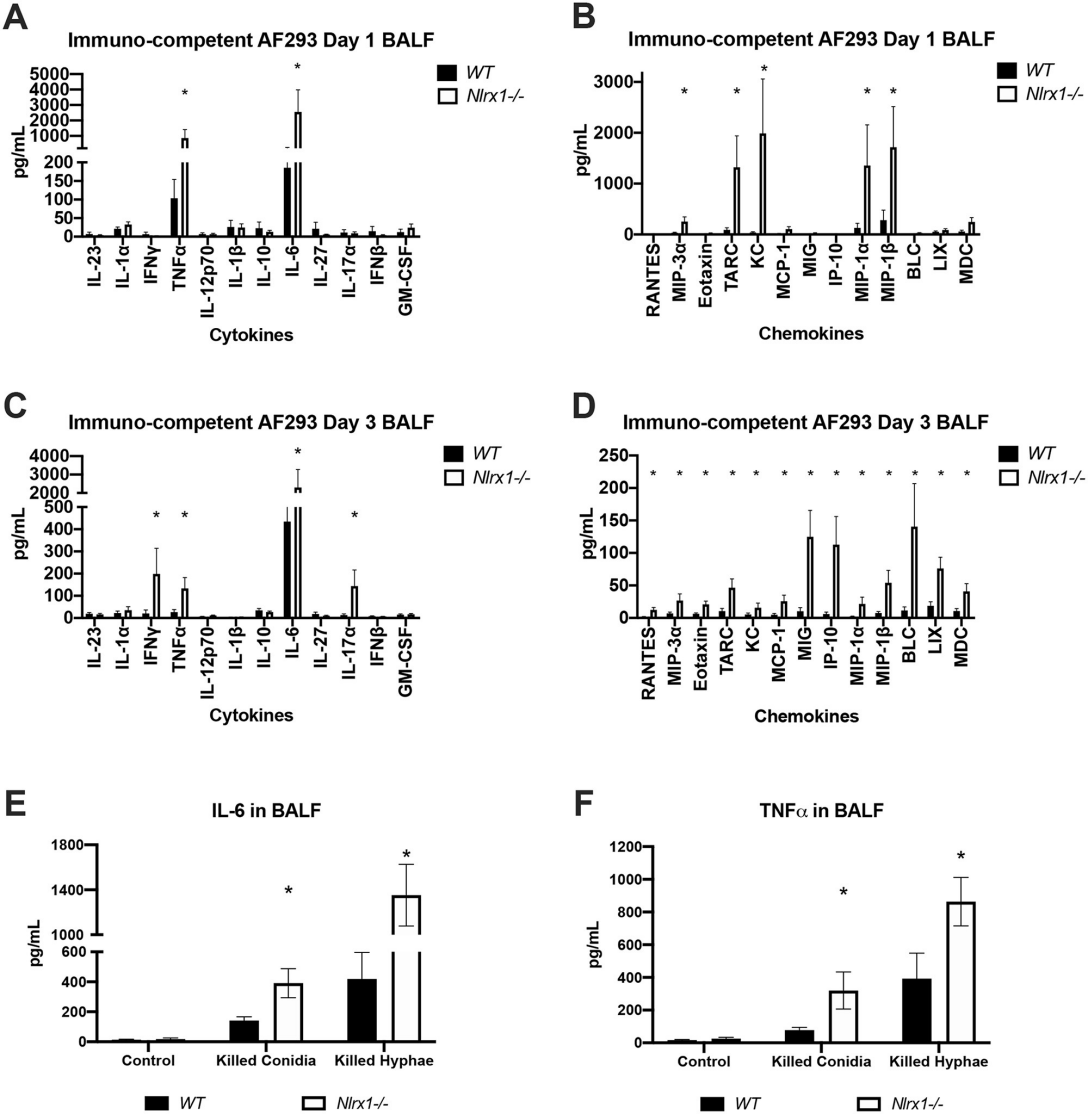
Characterization of secreted cytokines/chemokines and recruited leukocyte populations on day 1 and 3 post inoculation with AF293. Freshly harvested conidia from the AF293 isolate were delivered via aerosolization (12 mL, 1 X 10^9^ conidia/mL) to immuno-competent wild type and *Nlrx1-/-* mice. Bronchoalveolar lavage fluid (BALF) was extracted from mice on day 1 and 3 post inoculation. Concentration of (AC) cytokines (IL-23, IL-1α, IFNγ, TNFα, IL-12p70, IL-1β, IL-10, IL-6, IL-27, IL-17α, IFN-β, GM-CSF) and (BD) chemokines (RANTES (CCL5), MIP-3α (CCL20), Eotaxin (CCL1), TARC (CCL17), KC (CXCL1), MCP-1 (CCL2), MIG (CXCL9), IP-10 (CXCL10), MIP-1α (CCL3), MIP-1β (CCL4), BLC (CXCL13), LIX (CXCL5), MDC (CCL22)) in BALF on day 1 and 3 post inoculation was determined by flow cytometry bead based array. Measurement of (E) IL-6 and (F) TNFα in BALF 24 hours post challenge with killed conidia and hyphae and control PBS. (EF) Substrates delivered via non-invasive tracheal instillation. Asterisk denotes statistical significance, *P* < 0.05 Mann-Whitney U test. Error bars indicate standard deviation. N = 8–10 per experimental group.

We choose to determine if the enhanced PMN infiltrate and non-focal inflammation for *Nlrx1-/-* mice observed via H&E staining could be due to enhanced cytokine and chemokine signaling. We determined the concentration of 25 cytokines and chemokines in extracted BALF fluid from immuno-competent mice on day 1 and day 3 post aerosol inoculation with the AF293 isolate. We observed significantly elevated production of TNFα, IL-6, CCL20/MIP-3α, CCL17/TARC, CXCL1/KC, CCL3/MIP-1α, and CCL4/MIP-1β by *Nlrx1-/-* mice in response to the AF293 isolate on day 1 in comparison to wild type (*P* < 0.05, MWUT Fig 3A and 3B). Of these 7 cytokines/chemokines, only 4 were expressed in wild type at relatively low concentrations, while 3 (CCL20, CCL17, CXCL1) appeared to be uniquely expressed in *Nlrx1-/-* mice at this early stage of infection. Both MIP-3α and TARC are important chemokines for dendritic cell (DC) and T cell recruitment, while KC functions as a chemokine for neutrophils. By day 3 post inoculation elevated levels of 4 cytokines (IL-6, IFN-γ, IL-17a, TNF-α) and 13 chemokines (CCL5/RANTES, CCL20/MIP-3α, CCL11/Eotaxin, CCL17/TARC, CXCL1/KC, CCL2/MCP-1, CXCL9/MIG, CXCL10/IP-10, CCL3/MIP-1α, CCL4/MIP-1β, CXCL13/BLC, CXCL5/LIX, and CCL22/MDC) could be observed in *Nlrx1-/-* mice in comparison to wild type (*P* < 0.05, MWUT, Fig 3C and 3D). The elevated levels of inflammatory cytokines IL-6, IL-17a, IFN-γ and TNF-α correlate strongly with the enhanced pulmonary inflammation and cell death observed via H&E staining, LDH activity, and free albumin in BALF. Elevated chemokines in *Nlrx1-/-* mice were strongly associated with the recruitment and activation of macrophages (CXCL9, CCL4, CCL22), neutrophils (CXCL5, CXCL1, IL-17a, CCL3), monocytes (CXCL10, CCL2, CCL3, CCL22), eosinophils (CCL5, CCL11), natural killer cells (CCL17, CXCL9, CCL22), T-cell populations (CXCL10, CCL17, CXCL9, CCL4), DCs (CCL20), and B-lymphocytes (CXCL13). The loss of Nlrx1 resulted in a more diverse secretion of cytokines and chemokines that signal for the recruitment of leukocytes of the innate and adaptive immune response.

We became concerned that the enhanced inflammatory response was primarily due to the enhanced fungal burden observed across all pulmonary challenge models and not a loss of regulation in immune signaling towards the fungus. Measurement of IL-6 and TNFα in BALF 24 hours after challenge with killed conidia and killed hyphae indicated significantly increased production in *Nlrx1-/-* deficient mice in comparison to wild type (*P* < 0.05, MWUT, Fig 3E and 3F). This finding suggested that the Nlrx1 dependent hyper-inflammatory immune response is due to a loss of regulation in immune signaling that is then compounded by the enhanced fungal burden.

**Fig 4:**
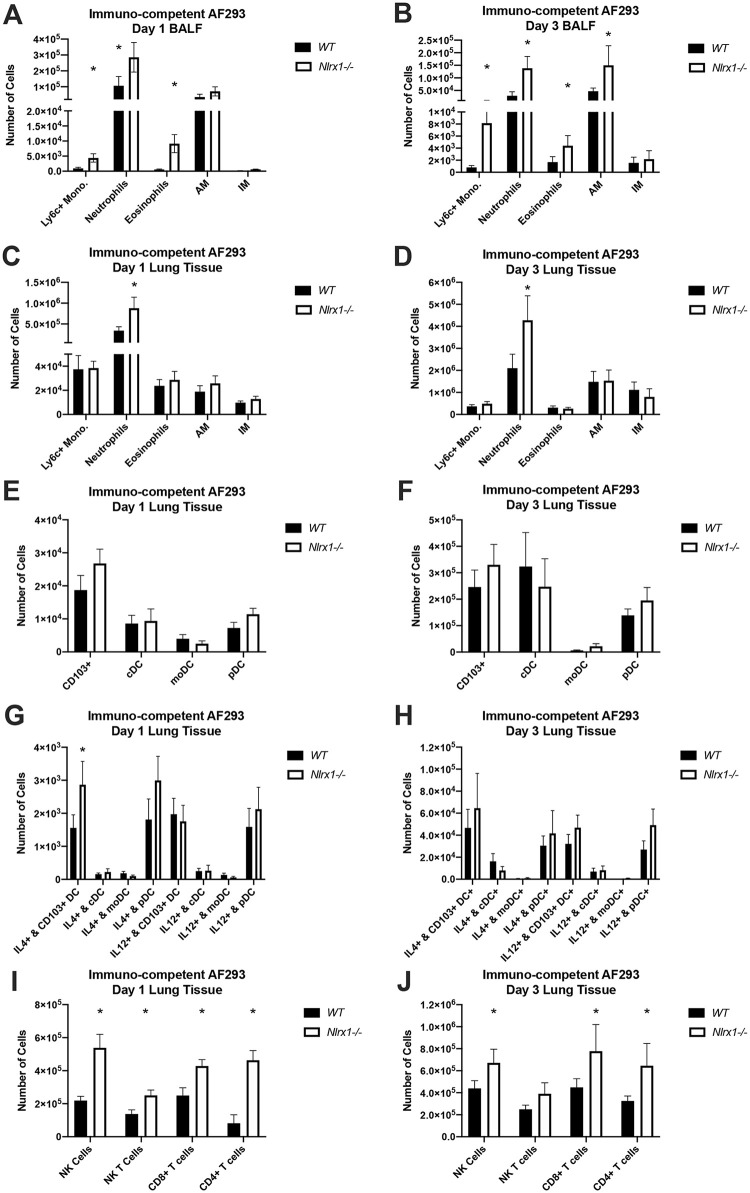
Analysis of recruited immune populations on day 1 and 3 post *A*. *fumigatus* challenge. Freshly harvested conidia from the AF293 isolate were delivered via aerosolization (12 mL, 1 X 10^9^ conidia/mL) to immuno-competent wild type and *Nlrx1-/-* mice. Recruited immune cell populations in (AB) bronchoalveolar lavage fluid BALF and (C-J) pulmonary tissue by wild type and *Nlrx1-/-* mice on day 1 and 3 post inoculation were determined by flow cytometry. Asterisk denotes statistical significance, *P* < 0.05 Mann-Whitney U test. Error bars indicate standard deviation. N = 8–10 per experimental group. AM, alveolar macrophages. IM, interstitial macrophages. DC, dendritic cells. pDC, plasmocytoid dendritic cells. moDC, monocytoid dendritic cells. cDC, conventional dendritic cells. NK, natural killer cells.

To quantify and determine the composition of immune cell recruitment, we analyzed BALF and pulmonary tissue infiltrate on days 1 and 3 post inoculation with the AF293 isolate from immuno-competent mice (gating strategy S2 Fig). The number of neutrophils, Ly6C+ monocytes, and eosinophils in BALF were significantly higher (doubling to an order of magnitude greater) in *Nlrx1-/-* mice in comparison to wild type on days 1 and 3 (*P* < 0.05, MWUT, Fig 4A and 4B). Further, three times as many alveolar macrophages could be observed on day 3 in *Nlrx1-/-* mice in comparison to wild type (*P* < 0.05, MWUT, Fig 4B). Of these five populations, only the recruitment of neutrophils in interstitial spaces was double for *Nlrx1-/-* mice in comparison to wild type on both days 1 and 3 (*P* < 0.05, MWUT, Fig 4C and 4D). Analysis of tissue specific dendritic cell populations indicated marginal increase in recruited CD103+, conventional (cDC), monocytoid (mDC), plasmacytoid (pDC) dendritic cell between the two genotypes (*P* > 0.10, MWUT, Fig 4E and 4F). Interestingly, the number of CD103+ dendritic cells producing IL-4 in *Nlrx1-/-* mice was double that of wild type on day 1, but not on day 3 (*P* < 0.05, MWUT, Fig 4G and 4H). Analysis of tissue specific T cell populations indicated a 2–4 fold increasing in natural killer (NK), CD8+ T cells, and CD4+ T cells for *Nlrx1-/-* mice (*P* < 0.05, MWUT, Fig 4I and 4J), though their intracellular cytokine production was consistent between genotypes (S4D and S4H Fig). The enhanced concentration of specific cytokines and chemokines observed in BALF by *Nlrx1-/-* mice correlated strongly with enhanced recruitment of their associated immune cell populations. Analysis of recruited leukocyte populations in BALF and tissue from immuno-competent mice on day 3 inoculated with the CEA10 isolate resulted in similar enhanced recruitment of neutrophils (BALF), and monocytes (tissue) (*P* < 0.05, MWUT, S5A and S5B Fig).

### Enhanced CD103+ IL-4+ DC recruitment correlates with enhanced *Nlrx1* mediated mortality

Given the observed effect of Nlrx1 dependent cytokine/chemokine production and immune cell recruitment in BALF and pulmonary tissue, we thought to determine if specific immune cell populations correlated with enhanced mortality and lung pathology. *Post-hoc* comparison of recruited immune cell populations in BALF and pulmonary tissue from wild type mice inoculated with either the AF293 or CEA10 strain indicated both general and specific depletions of immune cell populations driven primarily by immune status (S3–S7 Figs). Antibody induced Ly6G/Ly6C+ depletion resulted in a near complete depletion of neutrophils as well as partial depletion of DC populations, Ly6C+ monocytes, and NK T cells in comparison to immuno-competent mice during *A*. *fumigatus* challenge (S7 Fig). The chemotherapeutic model of IPA using the AF293 isolate indicated near complete depletion of T cell populations, NK cells, DC populations, eosinophils, AM and IMs in tissue. A partial depletion was also observed for monocytes and neutrophils in tissue samples, while the lavage cell counts were similar to immuno-competent level or further elevated. In general, studies with the CEA10 isolate resulted in enhanced immune cell recruitment in comparison to the AF293 isolate. In several instances, loss of Nlrx1 resulted in enhanced recruitment of specific immune cell populations unique to each immuno-suppressive regime suggesting it functions as a generic dampener of different forms of immune signaling (S3–S6 Figs).

**Fig 5:**
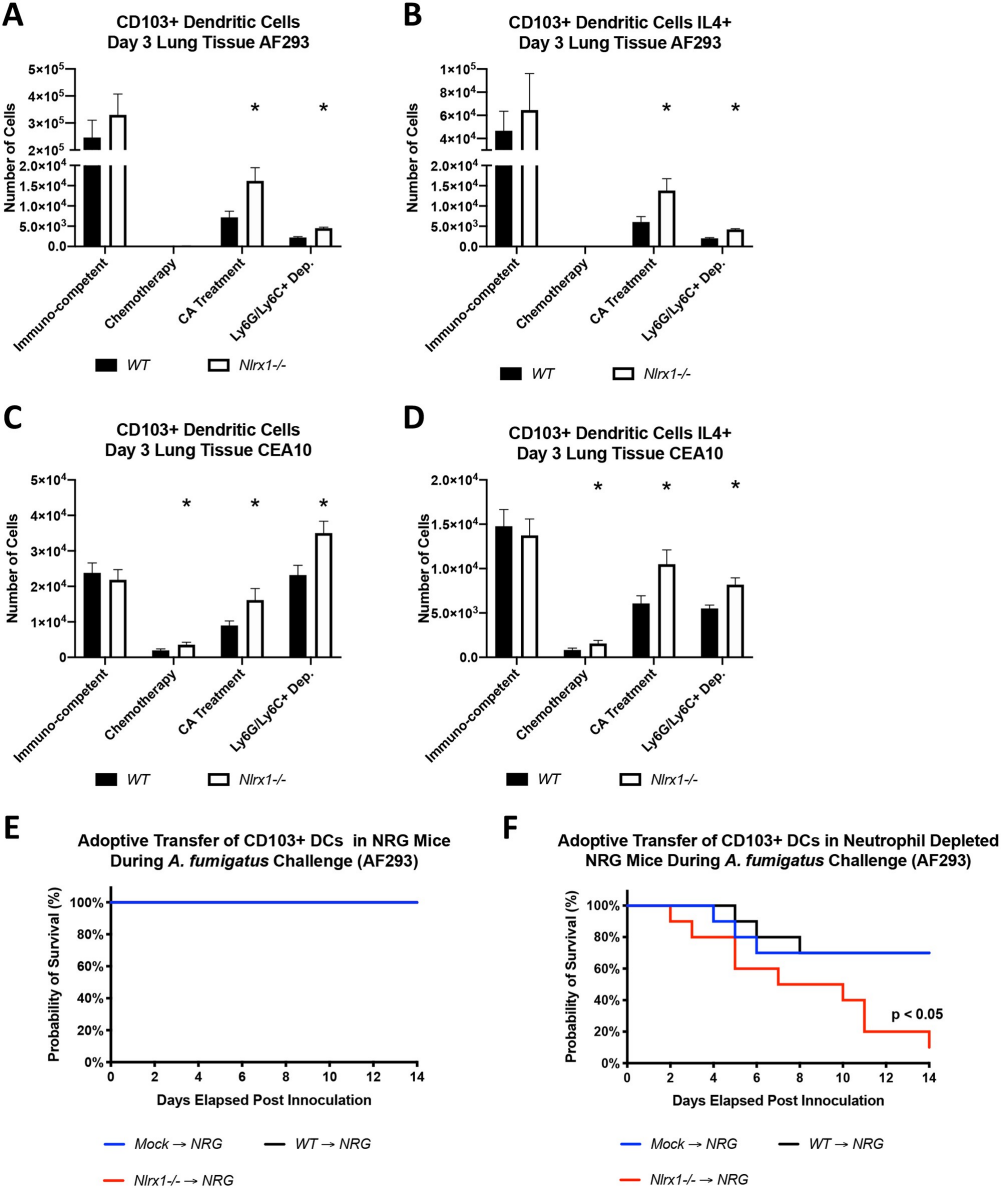
Characterization of pulmonary tissue dendritic cell populations and their adoptive transfer into NRG mice. (A-D) Freshly harvested conidia from either the AF293 or CEA10 isolate were delivered via aerosolization (12 mL, 1 X 10^9^ conidia/mL) to wild type and *Nlrx1-/-* mice under immuno-competent or immuno-suppressive conditions including antibody based induction of neutropenia (Ly6G/Ly6C+ depletion), cortisone acetate (CA) treatment, and chemically induced leukopenia (Chemotherapy). Mice were sacrificed on day 3 post inoculation and the number of (AC) CD103+ DCs and (BD) those producing IL-4 was determined via flow cytometry. (A-D) Asterisk denotes statistical significance, *P* < 0.05 Mann-Whitney U test. Error bars indicate standard deviation. N = 6–10. (E) Survival study post adoptive transfer of wild type and *Nlrx1-/-* CD103+ dendritic cells into NRG mice inoculated via non-invasive intratracheal instillation of AF293 conidia (10^7^ conidia/ 25μL). (F) Survival study post adoptive transfer of wild type and *Nlrx1-/-* CD103+ dendritic cells into NRG mice depleted of neutrophils via Ly6G+ depletion (1A8 Clone) one day prior and three days post inoculation. Inoculation occurred by non-invasive intratracheal instillation of AF293 conidia (10^7^ conidia/ 25μL). Statistical significance was determined using the log-rank (Mantel-Cox) test. N = 10 per experimental group.

Across all models where an increase in mortality for *Nlrx1-/-* mice was noted, an increase in both CD103+ dendritic cells populations and CD103+ DCs producing IL-4 was also observed (*P* < 0.05, MWUT, Fig 5A–5D). An increase in these populations was also noted in the chemotherapeutic model using the CEA10 isolate, but the increase should be viewed as negligible as greater than 90% of this population was depleted in comparison to the immuno-competent model (Fig 5C and 5D). It is important to also note that in 2 instances (Ly6G/Ly6C+ depletion and cortisone acetate treatment using the AF293 isolate) a 2-fold increase in *Nlrx1-/-* pDCs and *Nlrx1-/-* pDCs positive for IL-4 was also observed (*P* < 0.05, MWUT, S4J and S4N Fig). The elevated number of CD103+ DCs and CD103+ DCs producing IL-4, and in certain cases pDCs producing IL-4 correlated with enhanced mortality observed for both isolates.

### Adoptive transfer of *Nlrx1*-/- CD103+ DCs is detrimental to survival in the absence of neutrophils

Our survival studies and FACS analysis suggested a correlation with mortality and the occurrence of elevated CD103+ DC populations, specifically positive for IL-4 production. Whether CD103+ DCs positive for IL-4 production functioned as a marker or a detrimental factor during IPA remained to be addressed. To test the hypothesis that *Nlrx1-/-* CD103+ DCs are detrimental to survival during *A*. *fumigatus* challenge, we inoculated NRG mice with the AF293 isolate via non-invasive tracheal instillation and performed an adoptive transfer of wild type or *Nlrx1-/-* CD103+ DCs, or control media. NRG mice lack NK, B, T cells, and are defective in macrophages and dendritic cells. NRG mice receiving a mock adoptive transfer or adoptive transfer of wild type or *Nlrx1-/-* CD103+ DCs did not succumb to mortality (*P* > 0.10, Log-rank (Mantel-Cox) test, Fig 5E). This was not surprising as the innate immune system, particularly neutrophils, are believed to be fully functional in NRG mice. We then thought to deplete circulating neutrophils via antibody-based depletion using the neutrophil specific anti-mouse Ly6G antibody (clone 1A8) on day 1 prior to and on day 3 post inoculation. The adoptive transfer of *Nlrx1-/-* CD103+ DCs into NRG mice with neutropenia resulted in enhanced mortality in comparison to wild type and mock transfer (*P* < 0.05, Log-rank (Mantel-Cox) test, Fig 5F). Our data suggests that when neutrophils are depleted, CD103+ DCs are critical for survival during *A*. *fumigatus* pulmonary infection.

**Fig 6:**
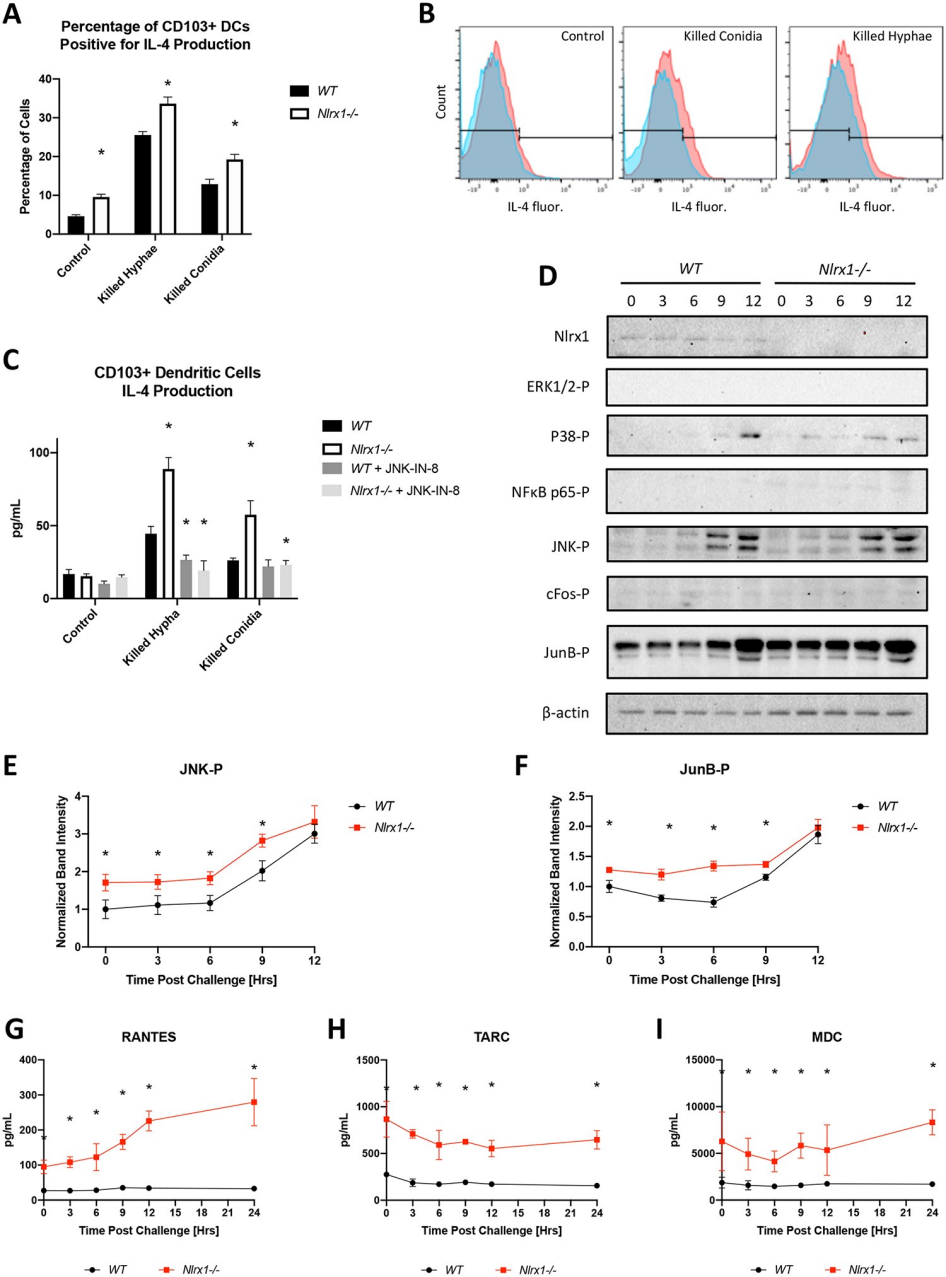
Temporal immune responses by CD103+ DCs during *in vitro* fungal challenge assays. Freshly harvested AF293 conidia (5 X 10^5^) were challenged against wild type and *Nlrx1-/-* CD103+ DCs (5 X 10^5^) at 37°C at 5% CO_2_. Supernatant and cells were harvested immediately prior to challenge (0 hrs), and at 3, 6, 9, 12 hrs post challenge. (A) Percentage of wild type and *Nlrx1-/-* CD103+ DCs staining positive for IL-4 12 hours post treatment with killed hyphae and conidia. (B) Representative histograms and gating for IL-4 fluorescence. See S2 Fig for strategy. (C) Intracellular measurement of IL-4 by wild type and *Nlrx1-/-* CD103+ dendritic cells 12 hours post treatment with killed hyphae and conidia in the presence and absence of the JNK inhibitor (JNK-IN-8). (D) Western blot analysis of phosphorylated (-P) ERK1/2-P, P38-P, NFκB p65-P, JNK-P, cFos-P, JunB-P, Nlrx1, and β-actin from wild type and *Nlrx1-/-* CD103+ dendritic cells during a 12-hour challenge against viable *A*. *fumigatus* conidia. Quantification of (E) JNK-P and (F) JunB-P. Measurement of secreted (G) RANTES (CCL5), (H) TARC (CCL17), (I) MDC (CCL22) by wild type and *Nlrx1-/-* CD103+ dendritic cells during a 24-hour challenge against viable *A*. *fumigatus* conidia. Asterisk denotes statistical significance, *P* < 0.05 Mann-Whitney U test. Error bars indicate standard deviation. N = 6–8 per experimental group.

Given that our *in vivo* results indicated an enhanced number of CD103+ DCs producing IL-4, we thought to determine if the loss of Nlrx1 in CD103+ DCs resulted in enhanced production of IL-4 in response to *A*. *fumigatus*. Using flow cytometry analysis of *in vitro* cultured CD103+ DCs we show that the absence of Nlrx1 results in a significantly greater percentage of cells (near 1.5-fold increase) positive for IL-4 production when challenged against killed conidia or hyphae (*P* < 0.05, MWUT, Fig 6A and 6B). Further, a significant percentage of control *Nlrx1-/-* CD103+ DCs are positive for IL-4 production suggesting these cells may be pre-primed towards a Th2 response (*P* < 0.05, MWUT, Fig 6A and 6B). Measurement of intra-cellular IL-4 by ELISA also indicated significantly enhanced IL-4 production in the absence of *Nlrx1-/-* when cells were challenged with killed conidia or hyphae (*P* < 0.05, MWUT, Fig 6C). Western blot analysis of CD103+ DCs challenged against viable *A*. *fumigatus* conidia over 12 hours showed enhanced phosphorylation of JNK and JunB, but not cFos, P38, or ERK1/2, for both wild type and *Nlrx1-/-* CD103+ DCs. The phosphorylation of JNK and JunB was significantly elevated in *Nlrx1-/-* CD103+ DCs prior to and post challenge when westerns blots were quantified suggesting Nlrx1 may also function as a negative regulator of this pathway (*P* < 0.05, MWUT, Fig 6D, 6E and 6F). Inhibition of JNK via the JNK Inhibitor XVI (JNK-IN-8) resulted in loss of IL-4 production as detected by ELISA for both genotypes (*P* < 0.05, MWUT, Fig 6C). This reiterated the effect of Nlrx1 on IL-4 production was due to its negative regulation of JNK. Additional analysis of secreted chemokines by CD103+ DCs in response to viable *A*. *fumigatus* conidia over 24 hours indicated enhanced secretion of RANTES (CCL5) and elevated basal signaling of TARC (CCL17) and MDC (CCL22) (*P* < 0.05, MWUT, Fig 6G, 6H and 6I). All three chemokines are strongly associated with the recruitment of Th2 polarized cells and eosinophils as observed *in vivo*.

**Fig 7:**
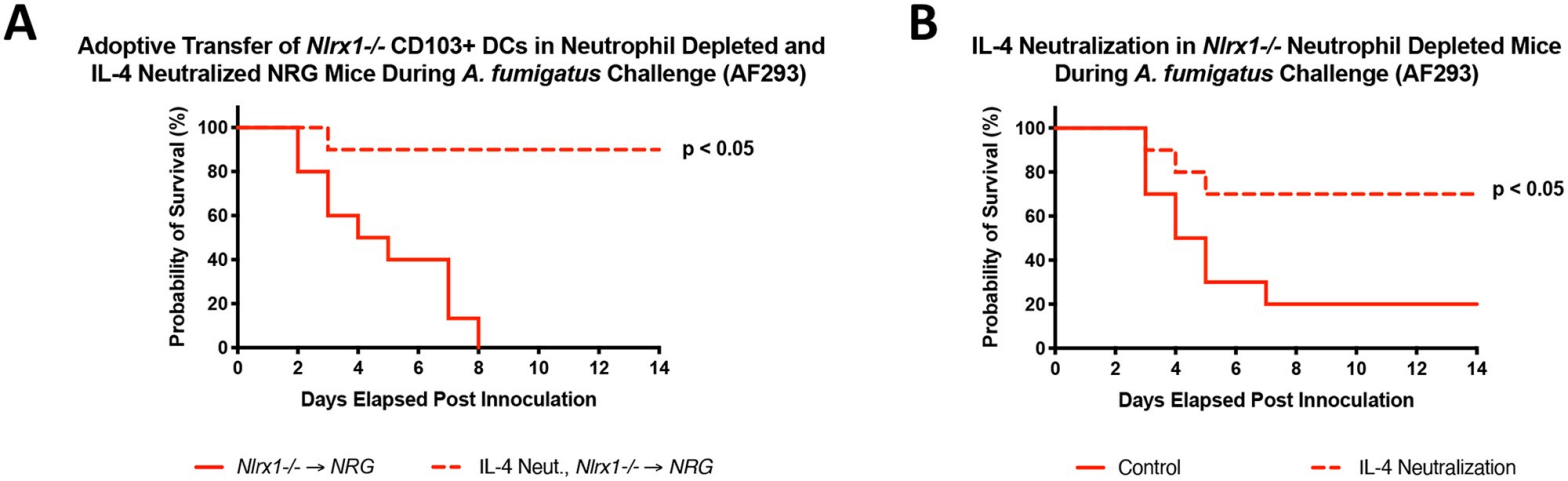
The effect of global IL-4 neutralization on survival during neutropenia models of invasive pulmonary aspergillosis using NRG and *Nlrx1-/-* mice. (A) Survival study post adoptive transfer of *Nlrx1-/-* CD103+ dendritic cells into NRG mice depleted of neutrophils via Ly6G+ depletion (1A8 Clone) and IL-4 neutralization (IL-4 Neut., *Nlrx1-/-* → NRG) or isotype control (*Nlrx1-/-* → NRG) antibody one day prior and three days post inoculation with AF293 conidia via non-invasive intratracheal instillation (10^7^ conidia/ 25μL). (B) Survival study for *Nlrx1-/-* mice depleted of neutrophils via Ly6G+ depletion (1A8 Clone) and IL-4 neutralization ((IL-4 Neut.) or isotype control antibody (Control) one day prior and three days post inoculation with AF293 conidia via non-invasive intratracheal instillation (10^7^ conidia/ 25μL). Statistical significance was determined using the log-rank (Mantel-Cox) test. N = 10 per experimental group.

Given the enhanced IL-4 production in the absence of Nlrx1 observed *in vivo* and *in vitro* in response to *A*. *fumigatus*, we thought to determine if depleting IL-4 would reduce mortality of *Nlrx1-/-* mice during relevant models of IPA. To test this hypothesis, we utilized an antibody depletion strategy to neutralize IL-4 one day prior to and three days post *A*. *fumigatus* inoculation during adoptive transfer of *Nlrx1-/-* CD103+ DCs into NRG mice undergoing neutrophil depletion. A near complete loss in mortality was observed when IL-4 was neutralized suggesting it was a primary agent of the observed mortality (*P* < 0.05, Log-rank (Mantel-Cox) test, Fig 7A). Similar experiments incorporating IL-4 neutralization in *Nlrx1-/-* deficient mice undergoing neutrophil depletion indicated enhanced survival post inoculation (*P* < 0.05, Log-rank (Mantel-Cox) test Fig 7B). We conclude that globally neutralizing IL-4 during these two specific models of IPA results in diminished mortality. However, we have yet to determine if the specific loss of IL-4 production by CD103+ DCs results in ablated mortality. This result would provide direct evidence that the enhanced mortality attributed to *Nlrx1-/-* CD103+ DC is due to their enhanced IL-4 production and leads to immunopathogenesis.

### Loss of *Nlrx1* in either radio-sensitive and insensitive cell populations impacts survival

**Fig 8:**
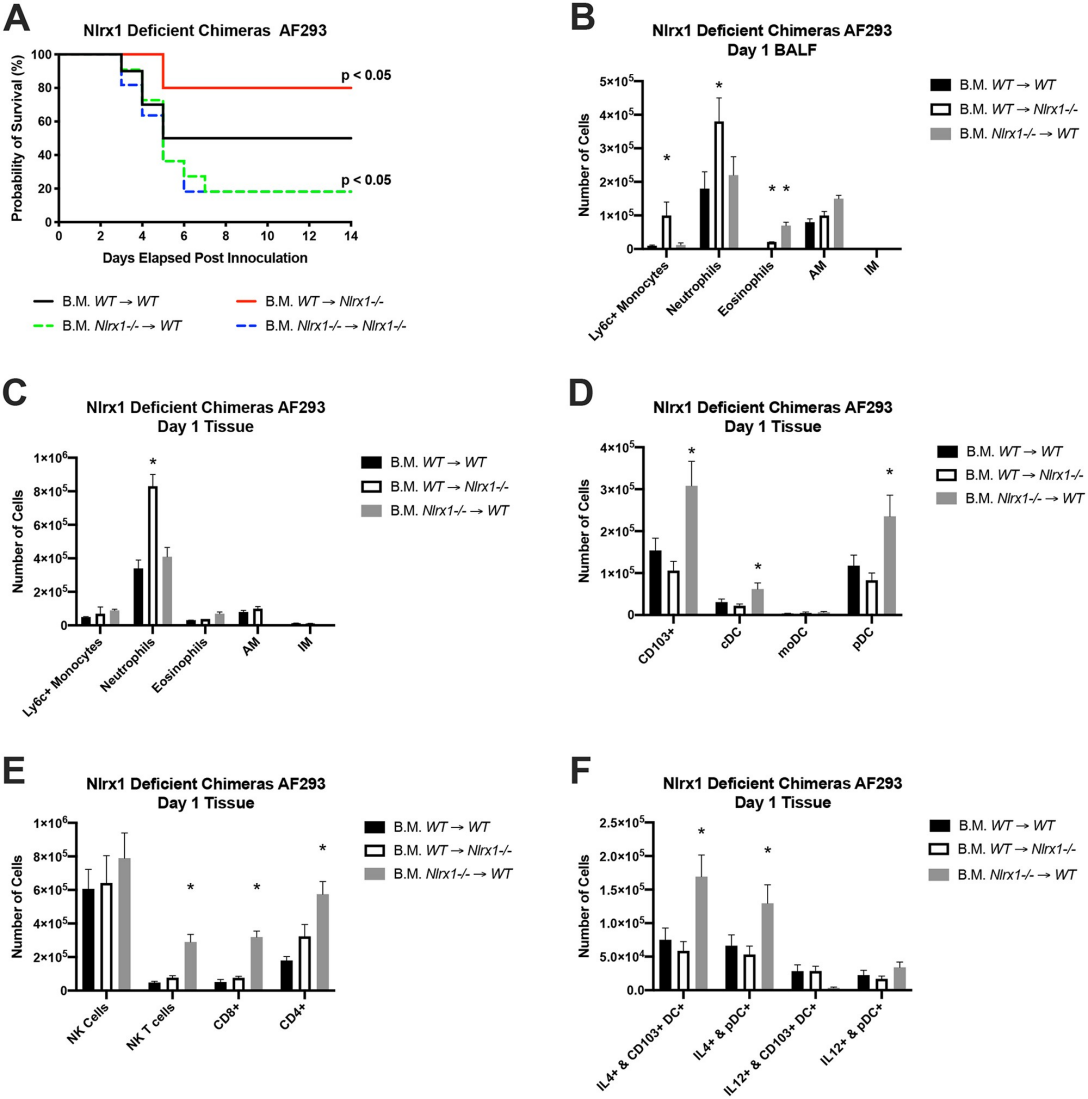
Loss of Nlrx1 in either radio-sensitive and insensitive cells populations impacts survival and immune cell recruitment. Wild type and *Nlrx1-/-* mice were irradiated and replenished with bone marrow from healthy wild type or *Nlrx1-/-* mice. Mice were allowed to recover for 6–8 weeks prior to inoculation. (A) Kaplan-Meier survival curve for neutrophil depleted chimeric mice inoculated with AF293 via non-invasive tracheal instillation AF293 conidia (10^7^ conidia/ 25μL). Mice were immuno-suppressed on day 1 prior and day 3 post inoculation using IP injection of Ly6G antibody (Clone A18). Statistical significance was determined using the log-rank (Mantel-Cox) test. N = 10 experimental group. (B-F) FACS analysis of immune cell populations from bronchoalveolar lavage fluid BALF and pulmonary tissue from immuno-competent chimeric mice on day 1 post inoculation were determined by FACS. Asterisk denotes statistical significance, *P* < 0.05 Mann-Whitney U test. Error bars indicate standard deviation. N = 8–10 per experimental group. AM, alveolar macrophages. IM, interstitial macrophages. DC, dendritic cells. pDC, plasmocytoid dendritic cells. moDC, monocytoid dendritic cells. cDC, conventional dendritic cells. NK, natural killer cells. NKT, natural killer T cells. CD8+, CD8+ T cells. CD4+, CD4+ T cells.

Given the importance of Nlrx1 in CD103+ DCs during models of *A*. *fumigatus* fungal challenge, we thought to utilize an adoptive transfer of bone marrow (B.M.) into irradiated mice to determine if the effects of *Nlrx1* on mortality, immune cell recruitment, and inflammation are attributed to its function in hematopoietic stem cells (radio-sensitive) and/or non-hematopoietic stem cells (radio-insensitive). Wild type and *Nlrx1-/-* mice were myeloablated via irradiation and reconstituted with bone marrow from wild type or *Nlrx1 -/-* mice within 24 hours. Mice were allowed to recover for 6–8 weeks, depleted of neutrophils on day -1 and day 3 post challenge, and challenged with 1 X 10^7^ AF293 conidia delivered through non-invasive tracheal instillation. A 14-day survival study indicated 50% survival for irradiated wild type mice reconstituted with wild type bone marrow (WT → WT) (Fig 8A). Approximately 80% of irradiated mice reconstituted with *Nlrx1-/-* bone marrow (*Nlrx1-/-* → WT and *Nlrx1-/-* → *Nlrx1-/-*) reached a moribund state within 6 days (*P* < 0.05, Log-rank (Mantel-Cox) test, Fig 8A). Contrastingly, *Nlrx1-/-* mice reconstituted with wild type bone marrow (WT → *Nlrx1-/-*) had an 80% survival rate over the 14-day study and a more gradual rate of decline (*P* < 0.05, Log-rank (Mantel-Cox) test, Fig 8A).

We then thought to look at immune cell populations in immuno-competent chimeric mice on day 1 post inoculations to determine if there were any significant variations amongst the chimeras. We observed enhanced recruitment of neutrophils, monocytes, and eosinophils in BALF for B.M. WT → *Nlrx1-/-* and eosinophils for B.M. *Nlrx1-/-* → WT mice in comparison to B.M. WT → WT mice (*P* < 0.05, MWUT, Fig 8B). Enhanced recruitment of neutrophils into lung tissue was also observed for B.M. WT → *Nlrx1-/-* mice (*P* < 0.05, MWUT, Fig 8C). Conversely, enhanced recruitment of CD103+ DCs, pDCs, cDCs, NK/NKT cells, CD4+ T cells, and CD8+ T cells occurred in B.M. *Nlrx1-/-* → WT mice in comparison to WT → WT mice (*P* < 0.05, MWUT, Fig 8D and 8E). Further a significantly larger number of CD103+ DCs and pDCs positive for IL-4 production were also observed for B.M. *Nlrx1-/-* → WT mice in comparison to WT → WT mice (*P* < 0.05, MWUT, Fig 8F). This finding fit well with our observation of elevated secretion of CCL5, CCL17, and CCL22 by CD103+ DCs *in vivo*. The enhanced mortality associated with *Nlrx1-/-* HSC populations was in accord with our findings on the detrimental role of *Nlrx1-/-* CD103+ DCs during IPA. Further the enhanced recruitment of T cell populations and eosinophils suggests a shift away from the innate immune response and a push towards a detrimental T2 response. Our results also indicate a novel synergy between *Nlrx1-/-* non-HSCs and wild type bone marrow that results in enhanced neutrophil recruitment critical for killing the fungus.

### *Nlrx1* deficient BEAS-2B cells have enhanced cytokine and novel chemokine production in response to *A*. *fumigatus*

**Fig 9:**
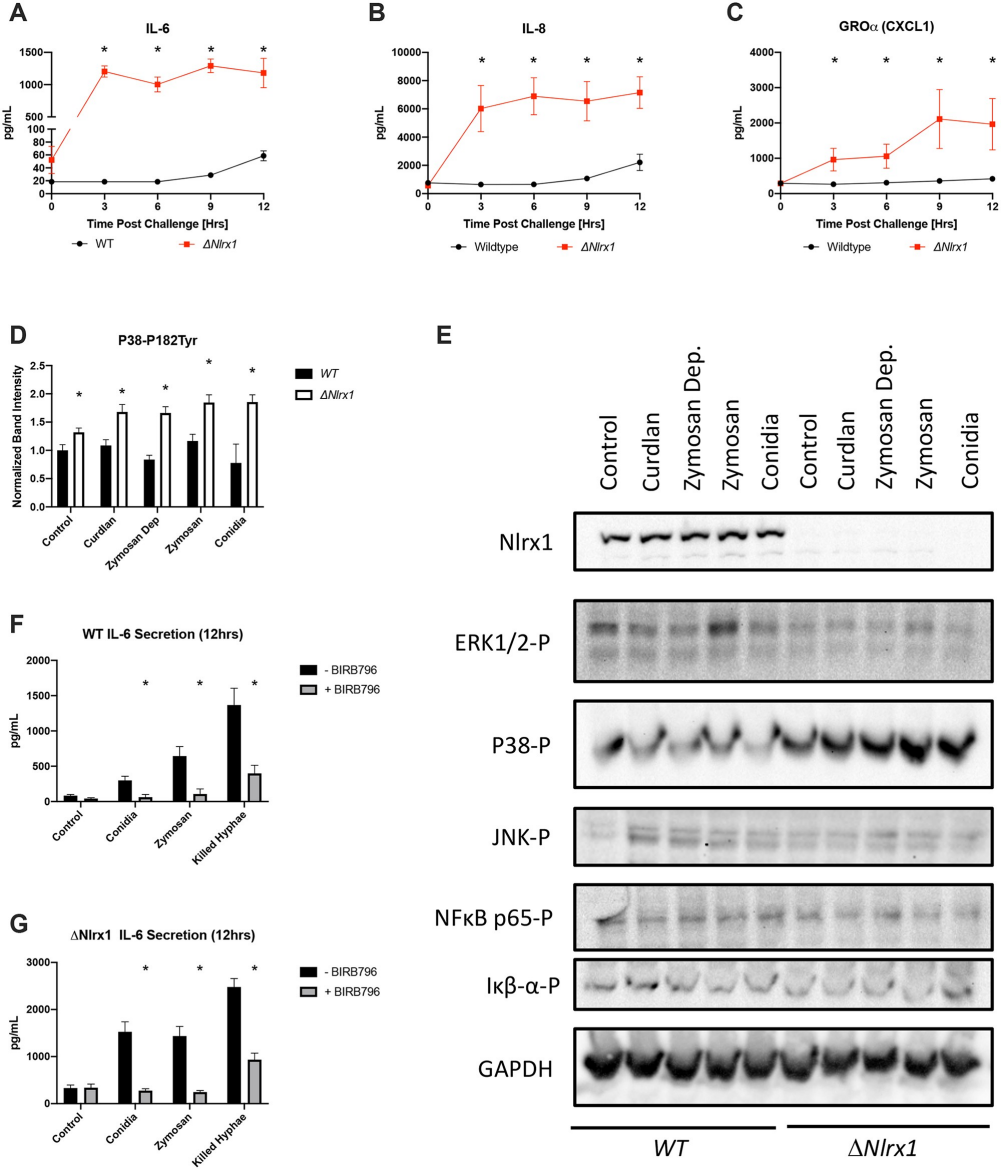
Temporal immune responses by BEAS-2B cells during *in vitro* fungal challenge assays. Freshly harvested AF293 conidia (5 X 10^5^) were challenged against wild type and ΔNlrx1 BEAS-2B airway epithelial cells (5 X 10^5^) at 37°C at 5% CO_2_. Total supernatant was harvested immediately prior to challenge (0 hrs), and at 3, 6, 9, 12 hrs post challenge. Concentration of cytokines (IL-1β, IFN-α, IFN-γ, TNF-α, MCP-1, IL-6, IL-8, IL-10, IL-12p70, IL-17A, IL-18, IL-23, and IL-33) and chemokines (MCP-1 (CCL2), RANTES (CCL5), IP-10 (CXCL10), Eotaxin (CCL11), TARC (CCL17), MIP-1α (CCL3), MIP-1β (CCL4), MIG (CXCL9), MIP-3α (CCL20), ENA-78 (CXCL5), GROα (CXCL1), I-TAC (CXCL11)) in culture media was determined by flow cytometry bead based array. (A-C) Differentially secreted cytokines and chemokines are presented. (DE) Western blot analysis and quantification of phosphorylated (-P) ERK1/2-P, P38-P, JNK-P, NFκB p65-P, Iκβ-α-P, Nlrx1, and GAPDH three hours post treatment with fungal PAMPs curdlan, zymosan, zymosan depleted, conidia, and control buffer. Independent experiments were run in triplicate. (FG) IL-6 secretion by wild type and ΔNlrx1 cells in response to conidia, zymosan, and hyphae at 12 hours in the presence and absence of P38 inhibitor BIRB796. Independent experiments were run in triplicate. All experiments were independently repeated, total N = 6. Error bars denote standard deviation. Statistical significance was determine using a Mann-Whitney U test and is indicated via asterisk, *P* < 0.05.

We became interested to determine which radio-insensitive cell populations were signaling for enhanced neutrophil and monocyte recruitment and inflammation in *Nlrx1-/-* mice and chimeric B.M. WT -> *Nlrx1-/-* mice in response to *A*. *fumigatus*. We hypothesized increased cytokine and chemokine secretion by airway epithelial cells would result in the enhanced immune cell recruitment. To test this hypothesis, we challenged wild type BEAS-2B cells, and Nlrx1 deficient BEAS-2B cells (ΔNlrx1) with AF293 conidia. We then assessed the temporal secretion of 25 cytokines and chemokines over a 12-hour challenge with viable *A*. *fumigatus* conidia. Wild type BEAS-2B cells began secreting significant amounts of IL-6 and CXCL8/IL-8 (neutrophil chemoattractant) from hours 6 through 12 post challenge, while ΔNlrx1 cells began producing 10–100 fold higher amounts of IL-6 and CXCL8 within 3 hours post challenge (*P* < 0.05, MWUT, Fig 9A and 9B). In the absence of Nlrx1, CXCL1/GROα (neutrophil chemoattractant) levels increased nearly 100-fold in comparison to wild type, which was not significantly secreted by wild type in response to *A*. *fumigatus* (*P* < 0.05, MWUT, Fig 9C). Significant, yet modest concentrations of CXCL11/I-TAC (T-cells), CXCL10/IP-10 (monocytes/macrophages, NK cells, T cells, DCs), CCL11/Eotaxin (eosinophils), and CXCL9/MIG (macrophages, NK cells, T cells) were observed for ΔNlrx1 cells challenged with AF293 conidia, but not wild type (*P* < 0.05, MWUT, S8 Fig). Temporal analysis suggested accumulation of secreted cytokines/chemokines by wild type BEAS-2B cells occurred at hours 9 and 12 post challenge, when swollen conidia are forming germlings and hypha. These same cytokines were detected in culture media by three hours post challenge against ΔNlrx1 cells. The substantially elevated and early secretion of IL-6, CXCL8, and CXCL1/GROα by ΔNlrx1 BEAS-2B cells in comparison to wild type correlated well with the enhanced neutrophil recruitment observed in BALF for *Nlrx1-/-* mice and *Nlrx1-/-* radio-resistant chimeras for the two genotypes as well as Nlrx1 dependent pulmonary inflammation.

We then set out to determine if the increased production of IL-6 and CXCL8 was mediated through a loss of negative regulation of immune signaling pathways by Nlrx1. Wild type and ΔNlrx1 cells were treated for 3 hours in the presence of fungal PAMPs and *A*. *fumigatus* viable conidia. Tested fungal PAMPs included Dectin-1 specific agonists curdlan and zymosan depleted, and Tlr2/Dectin-1 agonists zymosan. These three fungal PAMPs are commonly associated with elongating hyphae and not *A*. *fumigatus* conidia, which is primarily associated with Tlr4 mediated signaling. Probing total protein extracts using phospho-antibodies for P38, JNK, ERK1/2, and NFκB, and Iκβ-α via western blotting indicate enhanced phosphorylation of P38, but not NFκB, Iκβ-α, JNK or ERK1/2 in response to fungal PAMPs and *A*. *fumigatus* conidia for the ΔNlrx1 cells in comparison to wild type at 3 hours post treatment (Fig 9E). Quantification of P38 phosphorylation indicated an increased basal and nearly 2-fold increase of activated P38 (*P* < 0.05, MWUT, Fig 9D). These findings suggest Nlrx1 is a negative regulator of the P38 signaling pathway in response to fungal PAMPs and conidia by BEAS-2B cells. To further test this hypothesis, we pre-treated ΔNlrx1 and wild type cells with BIRB 796, a high affinity and selective P38 inhibitor, and challenged cells with fungal PAMP zymosan, *A*. *fumigatus* viable conidia and killed hyphae. IL-6 secretion was again significantly elevated in ΔNlrx1 cells in response to all three treatments and was nearly completed ablated when treated with BIRB 796 (*P* < 0.05, MWUT, Fig 9F and 9G). We conclude the early and hyper secretion of IL-6 and CXCL8 by ΔNlrx1 cells is due to a loss of negative regulation of P38 mediated pathways in response to conidia, killed hyphae, and fungal PAMPs. It will be important to determine if the enhanced cytokine production observed in the *in vitro* BEAS-2B model in the absence of Nlrx1 occurs for primary bronchial airway epithelial cells as this signaling may be responsible for the enhanced neutrophil recruitment observed *in vivo*.

## Discussion

The role of multi-faceted immune modulators, such as Nlrx1, provides a unique avenue to understand how biological processes such as immune signaling, metabolism, reactive oxygen species production and cell fate are interrelated in response to fungal infection. In our opinion this understanding is paramount to identifying viable host targeted therapeutics. We became interested in Nlrx1 due to it being amongst only a handful of Nlrs that we found to be differentially expressed during immuno-suppressive mouse models of IPA using the CEA10 isolate [28]. Nlrs, particularly those involved with the inflammasome, are important for a robust immune response to *A*. *fumigatus* [29–31]. Mice lacking both NLRP3 and AIM2 fail to confine *A*. *fumigatus*, leading to further growth and spreading to pulmonary blood vessels. This failure is associated with a lack of IL-1β and IL-18 secretion by BMDCs due to marginalized cytoplasmic inflammasome activation [29]. Conversely, loss of Nod1 in macrophages leads to enhanced conidial processing and ROS production by macrophages. *Nod1-/-* mice also have increased survival during immuno-suppressive models of IPA due to enhanced Dectin-1 expression and defense [32].

Our study revealed contrasting cell type-specific roles for Nlrx1 during IPA that significantly impacted mortality. Loss of Nlrx1 in CD103+ DCs resulted in increased IL-4 expression via enhanced Jun/JunB phosphorylation in response to *A*. *fumigatus*. This resulted in enhanced mortality for immuno-suppressive models of IPA where CD103+ DCs were an abundant population. Loss of Nlrx1 in non-hematopoietic stem cell populations resulted in enhanced inflammation and recruitment of innate immune cells through the hyper production of IL-6 and CXCL8/IL-8 family of interleukins respectively. This ultimately resulted in diminished mortality for *Nlrx1-/-* mice reconstituted with wild type bone marrow. *In vitro* models using BEAS-2B AECs highlighted the enhanced CXCL8, CXCL1, and IL-6 production is mediated by elevated P38 phosphorylation in the absence of Nlrx1. Nlrx1 appears to regulate critical and distinct aspects of the innate and adaptive immune responses in a cell type and pathway specific manner.

Nlrx1-mediated mortality occurred in a manner that was dependent on the type of immuno-suppression, while an increase in fungal burden occurred for all models in the absence of Nlrx1. Of all the analyzed immune cell populations only enhanced recruitment of CD103+ dendritic cells and those producing IL-4 correlated with mortality across all models. Further, adoptive transfer of *Nlrx1-/-* CD103+ DCs produced significantly enhanced mortality and enhanced IL-4 production *in vitro*. The enhanced mortality observed for these models was also ablated when IL-4 neutralizing antibodies were delivered. However, it is important to reiterate that IL-4 neutralization was global for these studies. The direct impact of loss of IL-4 in CD103+ DC on mortality has yet to be determined. There is a growing appreciating for the role DCs play during IPA due to their importance in shaping both the innate and adaptive immune response. Enhanced DC recruitment has been observed for neutropenia models of IPA suggesting their importance as an alternate line of defense [33]. Plasmocytoid DCs have been shown to play a critical non-redundant role during IPA via both fungal processing via secretion of extracellular traps as well as immune modulation [34, 35]. Inflammatory monocytoid DCs have been shown to differentiate into a terminal state from monocytes upon conidial uptake and this transition is critical for killing conidia [36]. Further, moDCs have been shown to shape the pulmonary inflammatory response to augment condia-cidal activity by neutrophils. CD103+ DCs have also been previously shown to control Th17 balance in the lungs via secretion of IL-2 [37]. A loss of IL-2 results in enhanced neutrophil influx in the lungs via excessive IL-23 production/secretion.

The loss of IL-4 production via *IL-4-/-* mice has shown to be beneficial during mouse models of IPA, and adoptive transfer of ex-vivo DCs primed with hyphae resulted in enhanced IL-4 production and their transfer into hosts is quite detrimental during IPA [38, 39]. Pulmonary DCs have been shown to produce large amounts of IL-4 in response to hyphae, but not conidia suggesting a morphological specific response [40]. IL-4 has previously been shown to be critical for the alternate activation of macrophages, recruitment and activation of eosinophils, and reduced functions of neutrophils during IPA [41–43]. Given the absence of T-cells in NRG mice we suspect IL-4 from CD103+ DCs is having a direct impact on these populations in lung tissue as well as shaping the overall T cell response. In the absence of Nlrx1 we have also observed enhanced eosinophil recruitment in BALF due to the enhanced production of CCL5, a potent chemokine for eosinophils, by CD103+ DCs [44]. *Nlrx1-/-* CD103+DC also produced enhanced levels of CCL22, a powerful chemokine for Th2 and Tc2 polarized cells expressing CCR4, and CCL17, a CD4+ lymphocyte chemokine that triggers IL-4 expression [45, 46]. Our findings advocate that the loss of Nlrx1 in CD103+ DCs primes them towards producing an excessive and detrimental Th2 response as well as a block aid of the Th17 response thereby enhancing mortality.

Loss of Nlrx1 results in enhanced inflammation and immune cell recruitment via specific cytokine/chemokine signaling. This signaling resulted in enhanced recruitment of primarily neutrophil, but also eosinophils, and T cell populations in immuno-competent mice. Neutralization of neutrophils and associated cytokines/chemokines (CXCL8/IL-8, CXCL1/KC) is associated with a detrimental effect on IPA [47, 48]. Similarly, depletion, knockout, or neutralization of IL-6 or TNFα is also detrimental to survival and fungal burden during IPA [49, 50]. The enhanced cytokine/chemokine signaling, and immune cell recruitment observed for *Nlrx1-/-* mice produced an interesting paradox as additionally recruited neutrophils, CD8+ T cells, and NK cells should aid in reducing fungal burden. A resolution to this paradox was suggested by our bone marrow transfer studies into irradiated mice. The adoptive transfer for wild type bone marrow into irradiated *Nlrx1-/-* mice resulted in significantly enhanced survival post *A*. *fumigatus* inoculation. Analysis of recruited immune cell populations indicated enhanced recruitment of neutrophils and monocytes, but not T cells and DC populations for B.M. WT → *Nlrx1-/-* mice. The enhanced recruitment of neutrophils and monocytes could be attributed to non-HSC population such as AECs, which are known to produce IL-6 and CXCL8 in response to *A*. *fumigatus* [51, 52]. Our results also indicated an enhanced mortality during IPA when bone marrow from *Nlrx1-/-* mice was transferred into WT mice. This fit well with our prior observations on the detrimental effect of *Nlrx1-/-* CD103+ DCs during IPA. Analysis of immune cell populations indicate that the influx of T cell populations and DCs for *Nlrx1-/-* mice is mediated by HSC populations. From a systems perspective our results suggest loss of Nlrx1 results in enhanced innate immune signaling between non-HSC, such as AECs and neutrophils. *Nlrx1-/-* HSC populations push a more robust adaptive response that is skewed towards a Th2 response.

CXCL8 production by airway epithelial cells occurs in part via ERK1/2 and P38 mediated pathways in response to swollen and germinating conidia [52]. We observed hyper phosphorylation of P38 as well as early and significantly enhanced IL-6 and CXCL8 production by ΔNlrx1 cells in comparison to WT in response to resting and germinating conidia in our *in vitro* BEAS-2B conidial challenge model. Though this finding correlated with the observed enhanced IL-6 and CXCL8 production in lavage fluid it is essential to note that enhanced IL-6 and CXCL8 production by primary airway epithelial cells lacking Nlrx1 has yet to be determined in a *in vivo* setting. Further, other cell populations, such as airway smooth muscle cells, may contribute to this phenomenon.

The use of the P38 kinase inhibitor prior to challenge against conidia resulted in diminished IL-6 production in the presence and absence of Nlrx1. P38 signaling is mediated in part by TRAF6, and Nlrx1 has previously been shown to be a negative regulator of TRAF6 mediated NF-κB activation in response to LSP treatment [19, 53]. We hypothesize that Nlrx1 may be functioning as a negative regulator of P38 activation in response to *A*. *fumigatus* resting conidia via sequestering TRAF6. Nlrx1 would then become inactivated in response to swollen conidia and hyphae resulting in P38 activation and IL-6/CXCL8/CXCL1 production. The role of Nlrx1 in mediating morphotype specific host responses will be fascinating to explore in the future.

An additional area of interest is the observation of cell specific regulation of P38 and JNK signaling pathways by Nlrx1 in BEAS-2B cells and CD103+ DCs respectively. IL-4 expression in Th cells occurs through JNK phosphorylation of JunB and c-Maf [54]. Our data indicates this pathway is utilized by dendritic cells for IL-4 production and is also negatively regulated by Nlrx1. Whether Nlrx1 regulation of JNK involves TRAF6 has yet to be determined, but is plausible given the TRAF6 mediates activation of JNK and P38 by TGF-β [55].

This study identifies the importance of host Nlrx1 during IPA for two contrasting yet clinically relevant isolates of *A*. *fumigatus*. We imagine Nlrx1 as a viable therapeutic target for invasive fungal infections given its role in modulating immune signaling and defense in responses to *A*. *fumigatus* in two distinct cell populations. We envision a scenario where immune cell recruitment and function could be amplified by inactivating Nlrx1 in non-HSCs. Conversely Nlrx1 may be manipulated in DC populations to mitigate IL-4 production. Identifying appropriate small molecule therapeutics targeting Nlrx1 may show efficacy in combating IPA and potentially other forms of pulmonary fungal disease.

## Methods

### Mouse models of pulmonary fungal challenge

Wild type and *Nlrx1-/-* C57/BL6 mice (7–12 weeks of age) were utilized for both fungal burden and survival studies in chemotherapeutic, Ly6G/C+ depletion, Ly6G+ depletion, and steroid treatment models of invasive pulmonary aspergillosis and immuno-competent model of pulmonary challenge. Mice were inoculated with resting conidia from *A*. *fumigatus* isolate AF293 or CEA10 using an aerosolization chamber as described in *Sheppard et al* [56] (2 exposures of 6 mL using a 1 x 10^9^ conidia / mL). Conidia were also delivered via non-invasive tracheal instillation (1 X 10^7^ conidia in 25 μL) for a subset of the studies where specified. In the chemotherapeutic model, mice were immunosuppressed 3 days prior to inoculation using an IP injection of cyclophosphamide (250 mg/kg) and a subcutaneous injection of cortisone acetate (250 mg/kg). Fungal burden and immune response were determined on day 3 post inoculation. For survival studies, mice received a second immuno-suppressive regime (IP injection of cyclophosphamide (200 mg/kg) and a subcutaneous injection of cortisone acetate (250 mg/kg) on day 3 post inoculation and were monitored a minimum of twice daily for 14 days post inoculation. In the Ly6G/Ly6C+ or Ly6G+ depletion model, mice were immunosuppressed 1 day prior to inoculation using an IP injection of RB6-8C5 or A18 antibody (80 μg/ 20 g) respectively. Fungal burden and immune response were determined on day 3 post inoculation. For survival studies mice received a second treatment of antibody on day 3 post inoculation and were monitored a minimum of twice daily for 14 days post inoculation. In the steroid model, mice were immunosuppressed 3 days prior to inoculation using a subcutaneous injection of cortisone acetate (250 mg/kg). Statistical significance for fungal burden studies was determined by the Mann-Whitney U test. Statistical significance for survival studies was determined by log-rank (Mantel-Cox) test.

For adoptive transfer in an irradiated mouse, mice were irradiated 4 days prior to inoculation with 2 doses of 900 rads approximately 6 hours apart. Within 18 hours mice were replenished with fresh extracted bone marrow from immuno-competent mice via tail vein injection. Mice were allowed to recover for 6–8 weeks and then inoculated with 1 X 10^7^ conidia in 25 μL via non-invasive tracheal instillation injection. Mice were monitored twice days for 14 days post inoculation. For adoptive transfer in NRG mice (*NOD*.*Cg-Rag1*^*tm1Mom*^ *Il2rg*^*tm1Wjl*^*/SzJ*), cultured wild type and *Nlrx1-/-* CD103+ DCs were injected via subcutaneous injection (5 X 10^6^ cells in 200 μL) post inoculation. For induction of neutropenia, a subcutaneous injection of Ly6G specific antibody (clone 1A8) was given one day prior to and three days post inoculation. For neutralization of IL-4, a subcutaneous injection of anti-IL-4 monoclonal antibody (200 μg/ 100 μL Clone 11B11) was given one day prior to and three days post inoculation. Mice were monitored twice daily for 14 days post inoculation. Statistical significance was determined by log-rank (Mantel-Cox) test.

### Fungal burden assay

Mouse lungs were harvested on day three post inoculation and homogenized by mortar and pestle under liquid nitrogen. 20 mg of total tissue was utilized for genomic DNA extraction using the Omega EZNA SP fungal DNA mini kit (D5542-02). Purified genomic DNA 500 ng was analyzed via qPCR using TaqMan assay specific for *A*. *fumigatus* 18s rDNA [57]. Forward Primer: 5’-GGCCCTTAAATAGCCCGGT-3’. Reverse Primer: 5’- TGAGCCGATAGTCCCCCTAA-3’. Taqman probe: 5’-/56-FAM/AGCCAGCGGCCCGCAAATG/3IABLFQ/-3’ (IDTDNA). Statistical difference was determined using a Mann-Whitney U test.

### Pulmonary infiltrate composition

To obtain and remove cells from the bronchoalveolar space, the trachea was cannulated postmortem using a gavage needle. Lungs were washed three times with 1 ml of room-temperature PBS that were subsequently combined. Protein transport inhibitor (BD #554724) was added after combination of three washes. Lungs inoculated with the CEA10 isolate were perfused with 5 mL of PBS. Lungs were chopped into small pieces and enzymatically digested at 37°C for 45 min. Red blood cells were eliminated by hypotonic lysis followed by filtration through a 70μM cell strainer. Remaining cells were resuspended in 1 mL of PBS containing 5% fetal bovine serum and 0.09% sodium azide. 6x10^5^ cells per well were plated into 96 well plates. Non-specific antibody binding was prevented through incubation with anti-CD16/anti-CD32 (BD #553142) prior to extracellular staining. Cells were incubated with a mixture of extracellular antibodies for 20 minutes at 4°C. Panel 1 contained: anti-CD45 (eBioscience #47-0451-82), anti-MHCII (eBioscience #13-5321-82), anti-CD11b (BD # 557960), anti-CD11c (BD #550261), anti-Ly6C (eBioscience #45-5932-82), anti-Gr1 (eBioscience #25-5931-82), anti-F4/80 (eBioscience #15-4801-82), anti-CD103 (BD #557494), and anti-SiglecF (BD #552126). Panel 2 contained: anti-CD45 (eBioscience #47-0451-82), anti-CD4 (eBioscience #56-0041-82), anti-CD8 (eBioscience # 45-0081-82), anti-CD3 (eBioscience #15-0031-83), anti-NK1.1 (eBioscience 25-5941-82). Panel 1 was incubated for 20 minutes with Streptavidin PE-CF594 (BD #562318) after centrifugation and wash. Panel 1 was then fixed in Cytofix Fixation Buffer (BD #554655). Panel 2 was fixed and permeabilized after extracellular staining with fixation/permeabilization buffer (eBioscience #00-5123-43 and 00-5223-56). Cells were incubated with intracellular antibodies, anti-IFNγ (BD #554412), anti-IL17 (BioLegend #506915), anti-IL12 (p40/p70) (BD #554480), and/or anti-IL4 (eBioscience #11-7042-82) with permeabilization buffer. Cell phenotype analysis was performed in FACS Diva with the following gating discrimination: 1) live cells based of FS vs SS, 2) doublet exclusion based on FSC-H vs FSC-W and SSC-H vs SSC-W, and 3) selection of CD45+ events. Thirty thousand CD45+ events were acquired with a LSRII flow cytometer (Becton Dickinson). CD45+ cells were then analyzed for cell phenotype. Flow cytometry gating strategy is shown in S2 Fig.

### Extraction and differentiation of CD103+ dendritic cells

Cells were extracted from *Nlrx1-/-* and wild type male mice between 15–25 weeks of age. Bone marrow derived CD103+ dendritic cells were cultured as previously described by [58]. In brief, cells were extracted from bone marrow in a sterile manner, subjected to hemolysis, filtered, washed in PBS and RMPI, and resuspended in RPMI media containing 10% FBS and Penn/Strep. Cells were diluted to a final concentration of 1 X 10^6^ cells / 1 mL. GM-CSF and FLT3L were added to final concentrations of 5 ng/mL and 200 ng/mL respectively and incubated for 9 days at 37°C in the presence of 5% carbon CO_2_. Cells were supplemented on day 5 with fresh media containing GM-CSF/FLT3L. On day 9, non-adherent/loosely adherent cells were harvest and plated in fresh media in either a 24-well format for *in vitro* studies or new T-75 flasks for *in vivo* studies. Cells were harvested on day 16 for use.

### Immunoassays for secreted chemokines and cytokines

Detection and quantification of secreted chemokines and cytokines were performed as described by the manufacturer (BioLegends, LEGENDplex, Mouse Inflammation Panel 740150, Mouse Proinflammatory Chemokine Panel 740007, Human Inflammation Panel 740118, Human Proinflammatory Chemokine Panel 740003). In brief, cell supernatant (500 μL) from pre- and post-challenge samples was harvested, centrifuged (10 min 5,000 x g), aliquoted (100 μL), and stored at -80°C. Procedures were conducted in a provided 96-well vacuum filtration plate using vacuum filtration device. Wells were pre-wet with 100 μL of 1X wash buffer for 1–2 min. A gentle vacuum was applied (5–10 sec) to drain buffer. Aliquots of samples (25 μL) or standards (25 μL) were incubated with 25 μL of assay buffer, and immune-bead solution (25 μL) for 2 hrs at room temperature (21–23 °C), shaking (~300 rpm) in the dark in a sealed plate. A gentle vacuum was applied to drain unbound molecules. Wells were washed with 200 μL of 1X wash buffer and a gentle vacuum was administered. The wash procedure was repeated. Detection antibody (25 μL) was added to each well and incubated at room temp, in the dark, for 1 hr shaking (~300 rpm) in a sealed plate. SA-PE (25 μL) was then added to each well and incubated at room temp in the dark, shaking (~300 rpm) in a sealed plate for 30 min. Samples were washed twice using 200 μL of 1X wash buffer and a gentle vacuum. Samples were suspended in 200 μL of 1X wash buffer and transferred to FACS analysis tubes containing 300 μL of 1X wash buffer. Samples were immediately analyzed via flow cytometry (LSR II). Approximately 8,000 gated events (large and small beads) were analyzed per sample or standard. Geometric mean fluorescence was determined for each distinct bead population using FLowJo(v10). Experiments were conducted in triplicate and repeated independently, N = 6–8. Statistical difference was determined using the Mann-Whitney U test.

### Western blot based analysis

Wild type and Nlrx1 deficient BEAS-2B airway epithelial cells and bone marrow derived CD103+ DCs (500,000 per well, 24-well format) were challenged with fungal PAMPs curdlan (100 ng/mL, InvivoGen), zymosan depleted (100 ng/mL, InvivoGen), zymosan (100 ng/mL, InvivoGen), and dead or living Af293 conidia (500,000 per well in 10 μL) for 3–12 hours at 37°C in the presence of 5% CO_2_ and harvested in NP-40 lysis buffer (250 μL) containing protease inhibitor cocktail and stored at -80°C until use. For specific experiments, cells were pre-incubated for 3 hours with BIRB 796 (100 nM) or JNK-IN-8 (10 nM). Loading Buffer (6x, 50 μL) was added to each sample and samples were heat denatured at 100°C for 10 minutes. Samples (20 μL) were loaded onto a 7.5% SDS-PAGE gel, run at 100V for 1.5 hrs and transferred onto nitro-celluloses using a wet transblotter (constant amperage at 0.35A, 1.5 hrs) in the presence on an ice block. Membranes were blocked in 3% BSA TBS-T and standard 3x wash procedure was followed. Primary, monoclonal antibodies were added at a concentration of (1:4000–1:20,000) and secondary antibodies were added at a concentration of (1:10,000–1:20,000). Membranes were developed using a Bio-rad ChemiDOC XRS+ with sequential exposure every 10 seconds from 10–600 seconds.

### ELISA

ELISA 96-well Plates for IL-6, CXCL8/IL-8, and IL-4 were setup and run according to the manufactures protocol (Biolegend). In brief, capture antibody was diluted in 100 μL of the diluted capture antibody solution and incubated overnight at 4 °C in the dark. Plates were then brought to room temperature and washed 4 times with a 0.05% PBS/Tween solution using an microplate washer. 200 μL of room-temperature 1X assay diluent A was then added to block non-specific binding. Plates were then sealed and incubated at room temperature while shaking. After the 1-hour incubation, the plate was again washed 4 times. Standards and samples were also added in triplicate. Plates were then sealed and incubated overnight in 4°C with shaking. Plates were then washed 4 times, and 100 μL of the diluted detection antibody solution was added to each well. Plates were then sealed and incubated at room temperature for 1 hour with shaking. After the incubation, plates were washed 4 times and 100 μL of the diluted avidin-HRP solution was added to each well. Plates were again sealed and incubated for 1 hour with shaking at room temperature. Plate were then washed 5 times and 100 μL of the substrate C solution was added to each well. Plates were incubated for 30 minutes in the dark and 100μL a stop solution of 2N H_2_SO_4_ was added. Plates were read at 450 nm and 570 nm within 30 minutes of adding the stop solution.

### Ethics statement

All animal studies were carried out in strict accordance with the recommendations in the Guide for the Care and Use of Laboratory Animals of the National Institute of Health. All protocols involving animals were approved by the institutional animal care and use committee (IACUC) and are in compliance with Public Health Service Policy (PHS). Approval Numbers: 16–085, 16–011, 16–130, 18–252, 19–013. Mice were humanely euthanized using fatal plus solution (immunological studies) or carbon dioxide induced narcosis followed by an approved secondary form of euthanasia.

## Supporting information

S1 Fig Mock and Control Survival Studies. Wild type and *Nlrx1-/-* mice under immuno-suppressive conditions including antibody-based induction of neutropenia (Ly6G/Ly6C+ depletion), cortisone acetate treatment, and chemically induced leukopenia (Chemotherapy) were mock aerosol inoculated and monitored for 14 days. (B) Immuno-competent wild type and *Nlrx1-/-* mice were aerosol inoculated with either the (B) the AF293 or (C) CEA10 isolate and monitored for 14 days. Statistical significance was determined using the log-rank (Mantel-Cox) test. (BC) N = 10 per experimental group. (A) Mock inoculation N = 5 per groups.
(TIF)

S2 Fig Flow Cytometry Gating Strategy. Four antibody panels were utilized to determine the identity of leukocyte populations. (A) Panel outline to determine percentage of CD45+ cells. (B) Gating strategy to identify alveolar macrophages, interstitial macrophages, neutrophils, eosinophils, and Ly6C+ monocytes. (C) Gating strategy to identify CD103+ dendritic cells, plasmocytoid dendritic cells, monocytoid dendritic cells, and conventional dendritic cells. Intra-cellular production of IL-4 and IL-12 is also determined for each cell population using Fluorescence minus one (FMO). (D) Gating strategy to identify natural killer cells, natural killer T cells, CD8+ T cells, and CD4+ T cells. Intra-cellular production of IL-4, IL-17a, and IFN-γ is also determined for each cell population.
(PDF)

S3 Fig Characterization of recruited leukocyte populations in BALF and pulmonary tissue on day 3 post inoculation with AF293. Freshly harvested AF293 conidia (12 X10^9^) were delivered via aerosolization to immuno-suppressive wild type and *Nlrx1-/-* mice. Three days post challenge recruited leukocyte populations in BALF and pulmonary tissue were characterized from wild type and *Nlrx1-/-* mice immuno-suppressed with (AB) antibody based induction of neutropenia (Ly6G/Ly6C+ depletion), (CD) cortisone acetate treatment, and (EF) chemically induced leukopenia (Chemotherapy). Asterisk denotes statistical significance, *P* < 0.05 Mann-Whitney U test. Error bars indicate standard deviation. N = 8–10 per experimental group. AM, alveolar macrophages. IM, interstitial macrophages.
(TIF)

S4 Fig Characterization of recruited interstitial dendritic cell and T cell populations their intra-cellular cytokine production post inoculation with AF293. Freshly harvested AF293 conidia (12 X10^9^) were delivered via aerosolization to immuno-competent and immuno-suppressive wild type and *Nlrx1-/-* mice. Recruited leukocyte populations in pulmonary tissue was determined for immuno-competent mice on (A-D) day 1 and (E-H) day 3 post inoculation. On day three post inoculation, recruited leukocyte populations in pulmonary tissue was determined for wild type and *Nlrx1-/-* mice immuno-suppressed with (I-L) antibody based induction of neutropenia (Ly6G/Ly6C+ depletion), (M-P) cortisone acetate treatment, and (Q-T) chemically induced leukopenia (Chemotherapy). Dendritic and T cell populations were stained for intracellular production of IL-12/IL-4 and IFN-γ/IL-17a/IL-4 respectively. Asterisk denotes statistical significance, *P* < 0.05 Mann-Whitney U test. Error bars indicate standard deviation. N = 8–10 per experimental group. CD103+, CD103+ dendritic cells. pDC, plasmocytoid dendritic cells. moDC, monocytoid dendritic cells. cDC, conventional dendritic cells. NK, natural killer cells. NKT, natural killer T cells. CD8+, CD8+ T cells. CD4+, CD4+ T cells.
(TIF)

S5 Fig Characterization of recruited leukocyte populations in BALF and pulmonary tissue on day 3 post inoculation with CEA10. Freshly harvested CEA10 conidia (12 X10^9^) were delivered via aerosolization to immuno-competent and immuno-suppressive wild type and *Nlrx1-/-* mice. Three days post challenge recruited leukocyte populations in BALF and pulmonary tissue were characterized from wild type and *Nlrx1-/-* mice (AB) immuno-competent or immuno-suppressed with (CD) antibody based induction of neutropenia (Ly6G/Ly6C+ depletion), (EF) cortisone acetate treatment, and (GH) chemically induced leukopenia (Chemotherapy). Asterisk denotes statistical significance, *P* < 0.05 Mann-Whitney U test. Error bars indicate standard deviation. N = 8–10 per experimental group. AM, alveolar macrophages. IM, interstitial macrophages.
(TIF)

S6 Fig Intra-cellular cytokine production by recruited dendritic and T cell populations in response to CEA10. Interstitial dendritic cell and T cell populations were identified on three days post challenge in wild type and *Nlrx1-/-* mice that were (A-D) immuno-competent or immuno-suppressed by (E-H) antibody based induction of neutropenia (Ly6G/Ly6C+ depletion), (I-L) cortisone acetate treatment, and (M-P) chemically induced leukopenia (Chemotherapy). Dendritic and T cell populations were stained for intracellular production of IL-12/IL-4 and IFN-γ/IL-17a/IL-4 respectively. N = 8. All experiments were independently repeated. Asterisk denotes statistical significance, *P* < 0.05 Mann-Whitney U test. Error bars indicate standard deviation. CD103+, CD103+ dendritic cells. pDC, plasmocytoid dendritic cells. moDC, monocytoid dendritic cells. cDC, conventional dendritic cells. NK, natural killer cells. NKT, natural killer T cells. CD8+, CD8+ T cells. CD4+, CD4+ T cells.
(TIF)

S7 Fig Re-plotting of infiltrated leukocyte populations from AF293 and CEA10 challenged mice. Indirect comparison of recruited leukocyte populations from immuno-competent or immuno-suppressed wild type mice on day 3 post inoculation with either the (A-D) AF293 or (E-H) CEA10 isolate. Mice were immuno-suppressed by antibody-based induction of neutropenia (Ly6G/Ly6C+ depletion), cortisone acetate treatment, and chemically induced leukopenia (Chemotherapy). AM, alveolar macrophages. IM, interstitial macrophages. CD103+ DC, CD103+ dendritic cells. pDC, plasmocytoid dendritic cells. moDC, monocytoid dendritic cells. cDC, conventional dendritic cells. NK, natural killer cells. NK T, natural killer T cells.
(TIF)

S8 Fig Cytokine and chemokine secretion by wild type and Nlrx1 deficient BEAS-2B cells in response to *A*. *fumigatus* conidia. Freshly harvested AF293 conidia (5 X 10^5^) were challenged against wild type and ΔNlrx1 BEAS-2B airway epithelial cells (5 X 10^5^) at 37°C at 5% CO_2_. Total supernatant was harvested immediately prior to challenge (0 hrs), and at 3, 6, 9, 12 hrs post challenge. Concentration of (A) CXCL11/I-TAC, (B) CXCL10/IP-10, (C) CCL11/Eotaxin, and (D) CXCL9/MIG in culture supernatant.
(TIF)
