# Supplementary figures and images for: NLRX1 is a key regulator of immune signaling during invasive pulmonary aspergillosis
Source: PLoS Pathog. 2020 Sep 21;16(9):e1008854. doi: 10.1371/journal.ppat.1008854 (PMC7529209; doi:10.1371/journal.ppat.1008854)

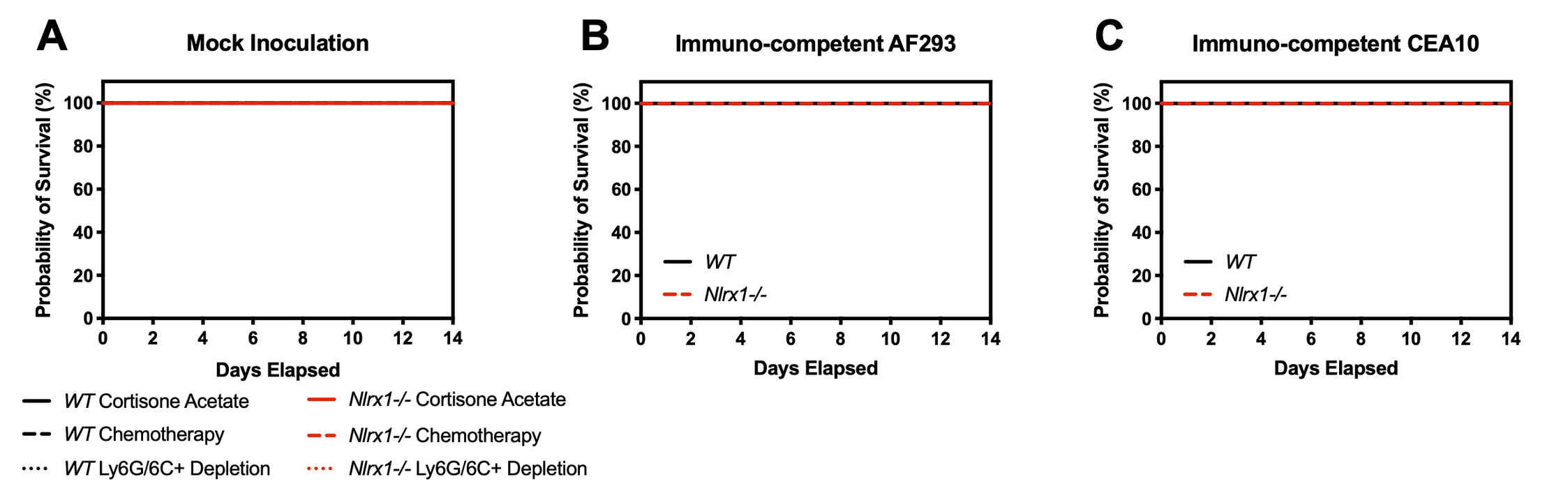

Supplement: S1 Fig — Wild type and Nlrx1-/- mice under immuno-suppressive conditions including antibody-based induction of neutropenia (Ly6G/Ly6C+ depletion), cortisone acetate treatment, and chemically induced leukopenia (Chemotherapy) were mock aerosol inoculated and monitored for 14 days. (B) Immuno-competent wild type and Nlrx1-/- mice were aerosol inoculated with either the (B) the AF293 or (C) CEA10 isolate and monitored for 14 days. Statistical significance was determined using the log-rank (Mantel-Cox) test. (BC) N = 10 per experimental group. (A) Mock inoculation N = 5 per groups. (TIF) [file ppat.1008854.s001.tif]

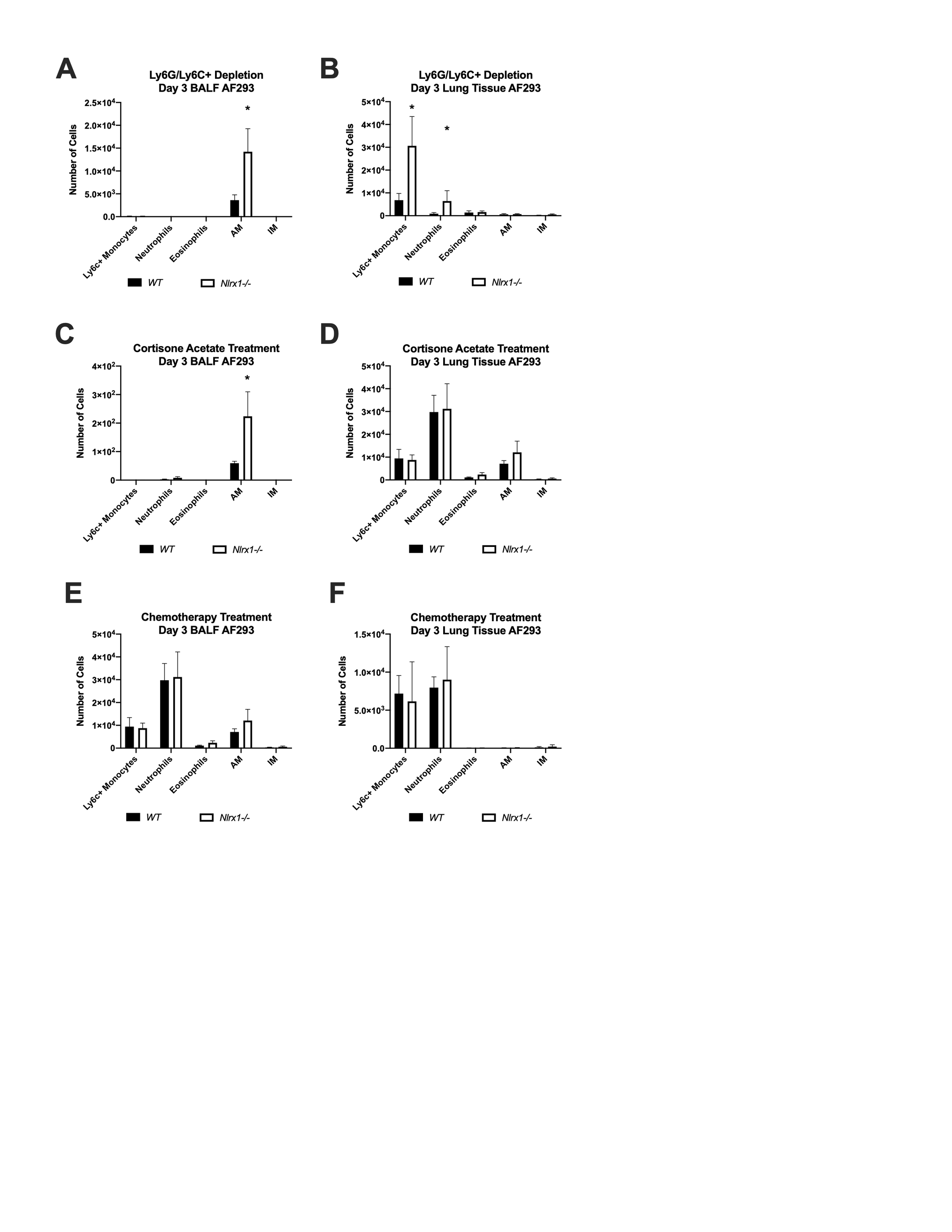

Supplement: S3 Fig — Freshly harvested AF293 conidia (12 X109) were delivered via aerosolization to immuno-suppressive wild type and Nlrx1-/- mice. Three days post challenge recruited leukocyte populations in BALF and pulmonary tissue were characterized from wild type and Nlrx1-/- mice immuno-suppressed with (AB) antibody based induction of neutropenia (Ly6G/Ly6C+ depletion), (CD) cortisone acetate treatment, and (EF) chemically induced leukopenia (Chemotherapy). Asterisk denotes statistical significance, P < 0.05 Mann-Whitney U test. Error bars indicate standard deviation. N = 8–10 per experimental group. AM, alveolar macrophages. IM, interstitial macrophages. (TIF) [file ppat.1008854.s003.tif]

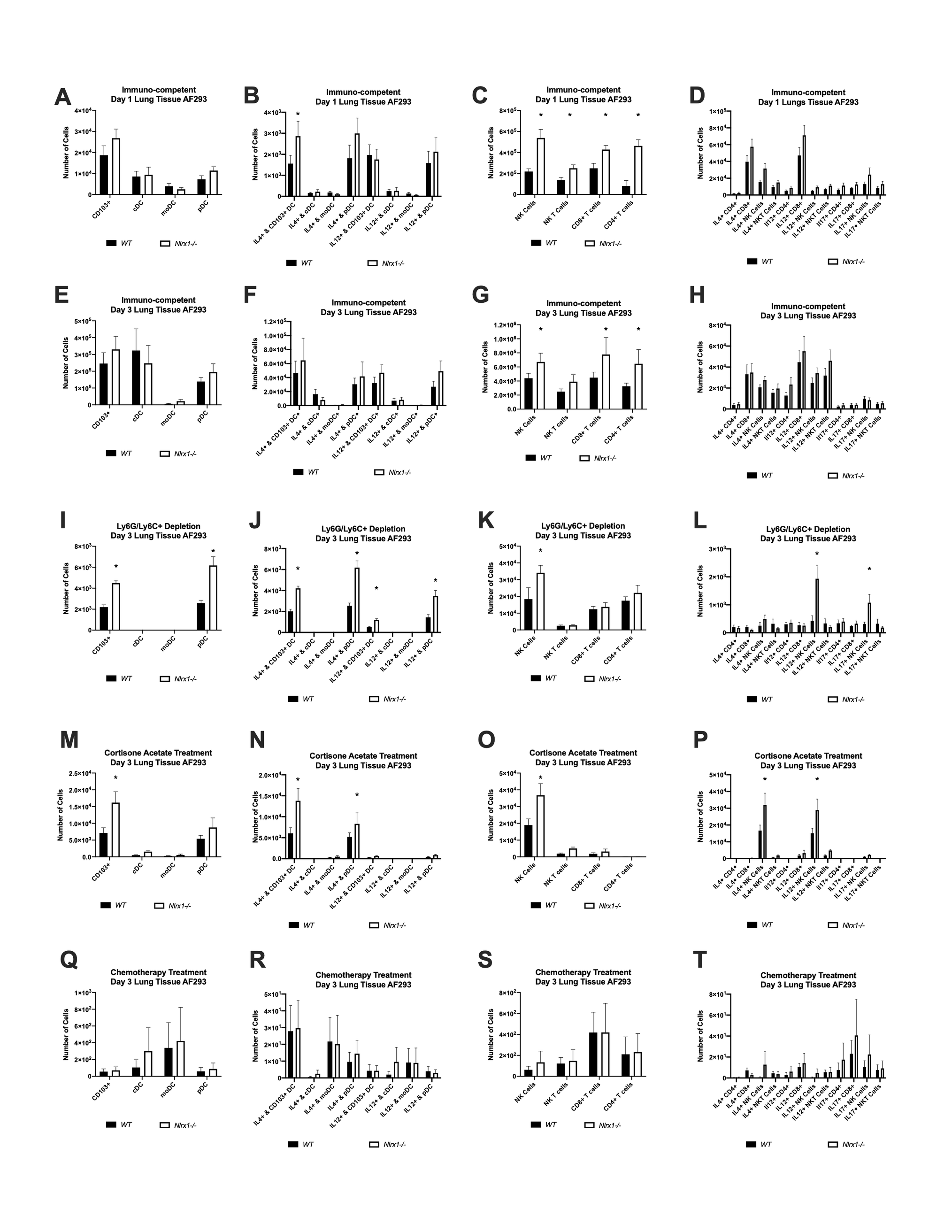

Supplement: S4 Fig — Freshly harvested AF293 conidia (12 X109) were delivered via aerosolization to immuno-competent and immuno-suppressive wild type and Nlrx1-/- mice. Recruited leukocyte populations in pulmonary tissue was determined for immuno-competent mice on (A-D) day 1 and (E-H) day 3 post inoculation. On day three post inoculation, recruited leukocyte populations in pulmonary tissue was determined for wild type and Nlrx1-/- mice immuno-suppressed with (I-L) antibody based induction of neutropenia (Ly6G/Ly6C+ depletion), (M-P) cortisone acetate treatment, and (Q-T) chemically induced leukopenia (Chemotherapy). Dendritic and T cell populations were stained for intracellular production of IL-12/IL-4 and IFN-γ/IL-17a/IL-4 respectively. Asterisk denotes statistical significance, P < 0.05 Mann-Whitney U test. Error bars indicate standard deviation. N = 8–10 per experimental group. CD103+, CD103+ dendritic cells. pDC, plasmocytoid dendritic cells. moDC, monocytoid dendritic cells. cDC, conventional dendritic cells. NK, natural killer cells. NKT, natural killer T cells. CD8+, CD8+ T cells. CD4+, CD4+ T cells. (TIF) [file ppat.1008854.s004.tif]

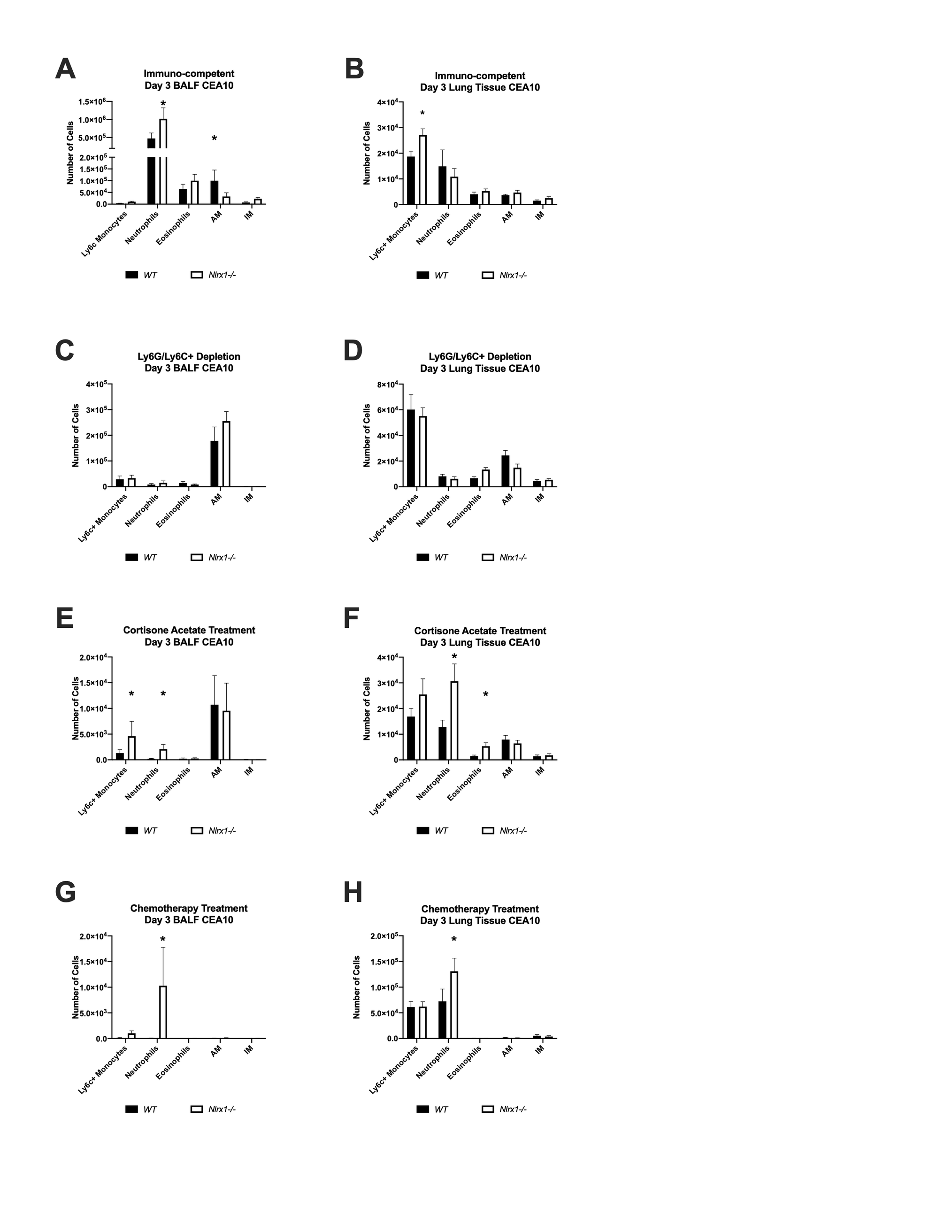

Supplement: S5 Fig — Freshly harvested CEA10 conidia (12 X109) were delivered via aerosolization to immuno-competent and immuno-suppressive wild type and Nlrx1-/- mice. Three days post challenge recruited leukocyte populations in BALF and pulmonary tissue were characterized from wild type and Nlrx1-/- mice (AB) immuno-competent or immuno-suppressed with (CD) antibody based induction of neutropenia (Ly6G/Ly6C+ depletion), (EF) cortisone acetate treatment, and (GH) chemically induced leukopenia (Chemotherapy). Asterisk denotes statistical significance, P < 0.05 Mann-Whitney U test. Error bars indicate standard deviation. N = 8–10 per experimental group. AM, alveolar macrophages. IM, interstitial macrophages. (TIF) [file ppat.1008854.s005.tif]

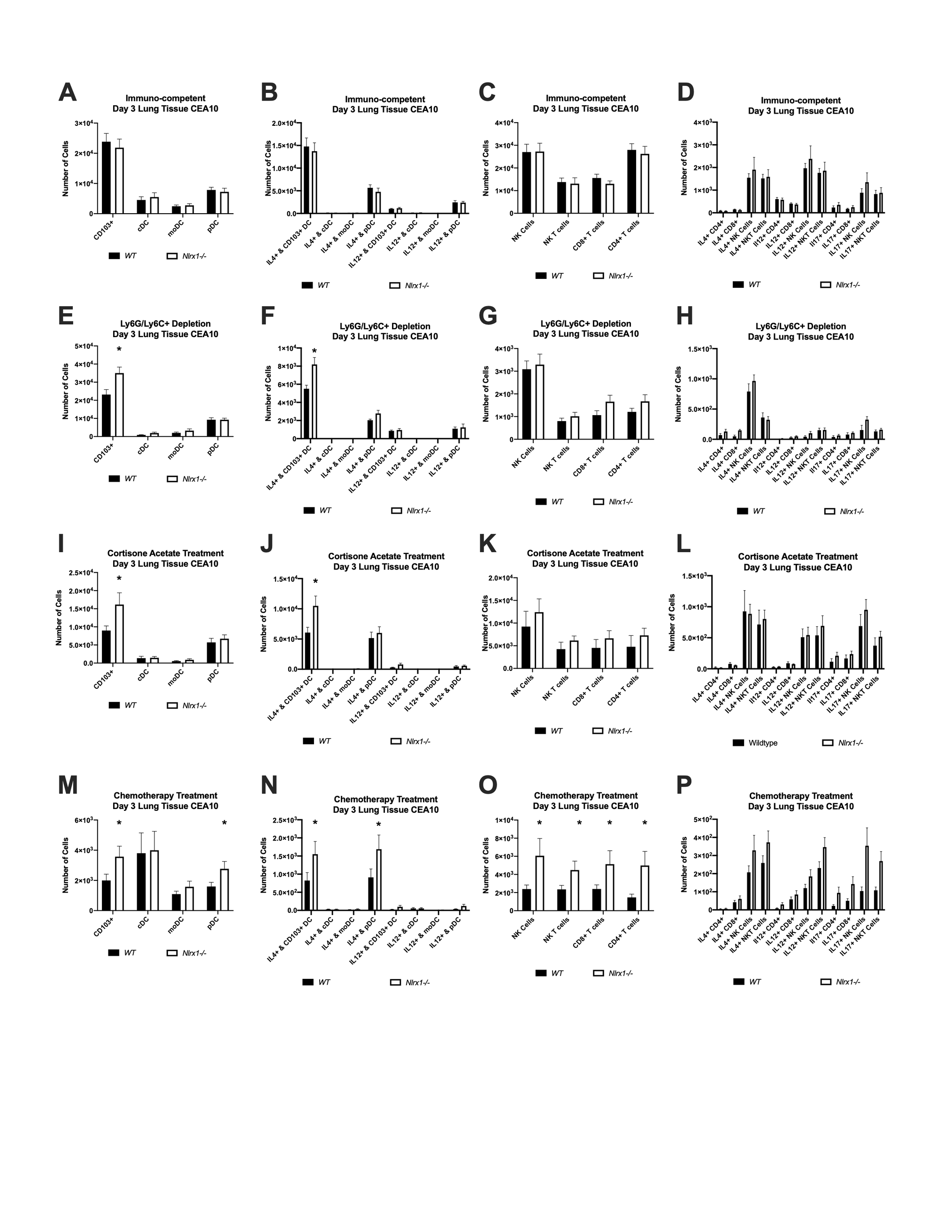

Supplement: S6 Fig — Interstitial dendritic cell and T cell populations were identified on three days post challenge in wild type and Nlrx1-/- mice that were (A-D) immuno-competent or immuno-suppressed by (E-H) antibody based induction of neutropenia (Ly6G/Ly6C+ depletion), (I-L) cortisone acetate treatment, and (M-P) chemically induced leukopenia (Chemotherapy). Dendritic and T cell populations were stained for intracellular production of IL-12/IL-4 and IFN-γ/IL-17a/IL-4 respectively. N = 8. All experiments were independently repeated. Asterisk denotes statistical significance, P < 0.05 Mann-Whitney U test. Error bars indicate standard deviation. CD103+, CD103+ dendritic cells. pDC, plasmocytoid dendritic cells. moDC, monocytoid dendritic cells. cDC, conventional dendritic cells. NK, natural killer cells. NKT, natural killer T cells. CD8+, CD8+ T cells. CD4+, CD4+ T cells. (TIF) [file ppat.1008854.s006.tif]

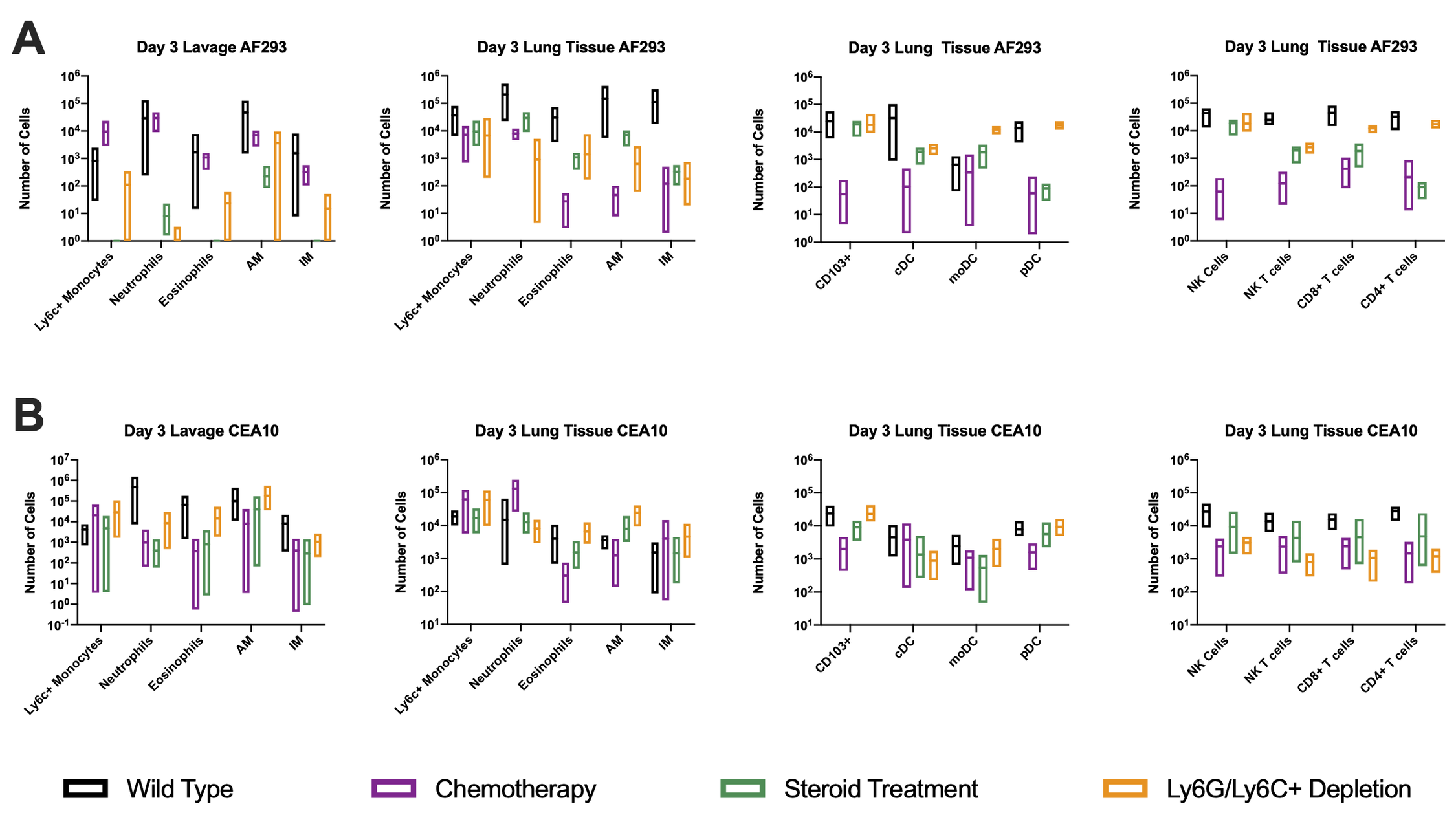

Supplement: S7 Fig — Indirect comparison of recruited leukocyte populations from immuno-competent or immuno-suppressed wild type mice on day 3 post inoculation with either the (A-D) AF293 or (E-H) CEA10 isolate. Mice were immuno-suppressed by antibody-based induction of neutropenia (Ly6G/Ly6C+ depletion), cortisone acetate treatment, and chemically induced leukopenia (Chemotherapy). AM, alveolar macrophages. IM, interstitial macrophages. CD103+ DC, CD103+ dendritic cells. pDC, plasmocytoid dendritic cells. moDC, monocytoid dendritic cells. cDC, conventional dendritic cells. NK, natural killer cells. NK T, natural killer T cells. (TIF) [file ppat.1008854.s007.tif]

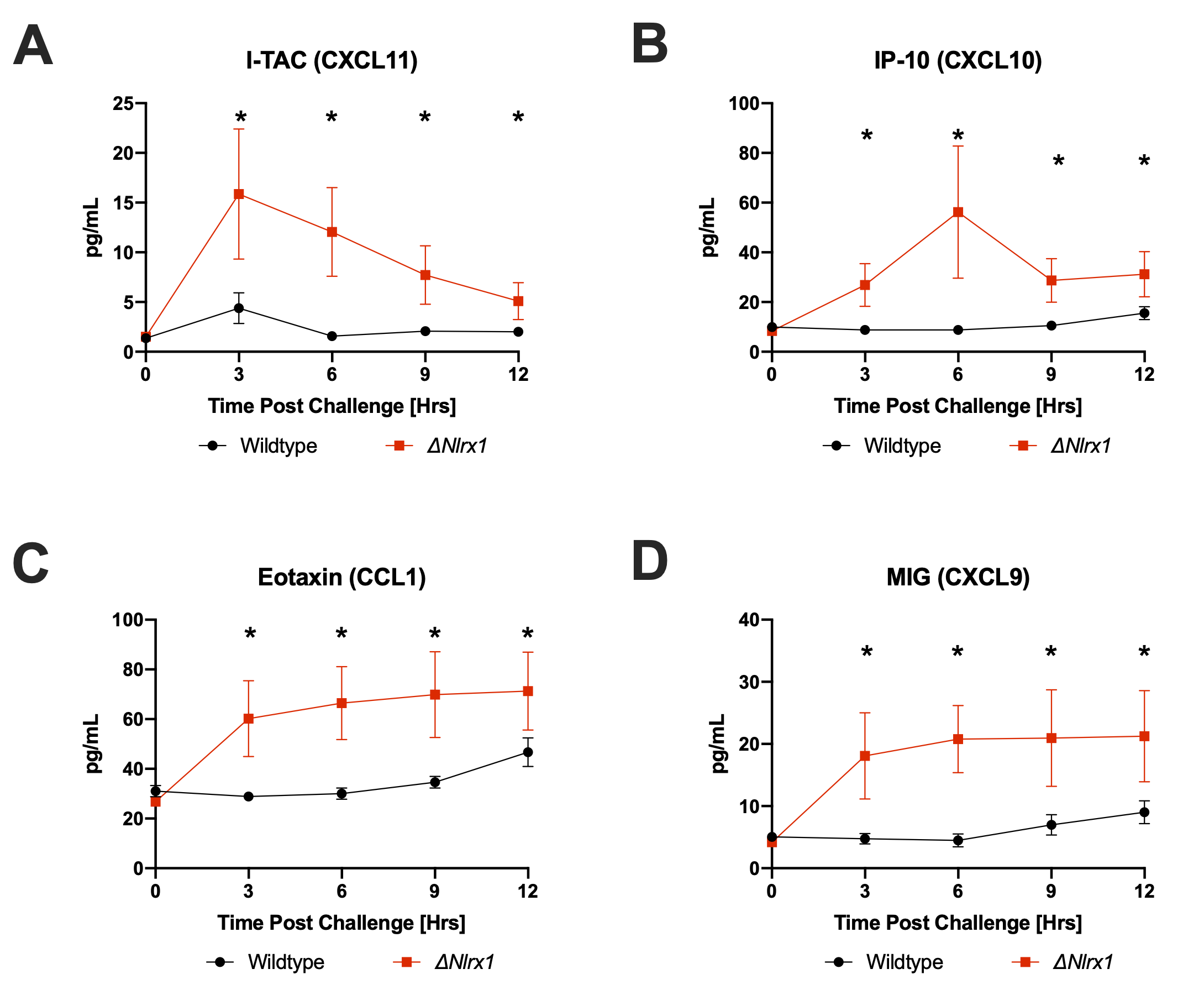

Supplement: S8 Fig — Freshly harvested AF293 conidia (5 X 105) were challenged against wild type and ΔNlrx1 BEAS-2B airway epithelial cells (5 X 105) at 37°C at 5% CO2. Total supernatant was harvested immediately prior to challenge (0 hrs), and at 3, 6, 9, 12 hrs post challenge. Concentration of (A) CXCL11/I-TAC, (B) CXCL10/IP-10, (C) CCL11/Eotaxin, and (D) CXCL9/MIG in culture supernatant. (TIF) [file ppat.1008854.s008.tif]
