# Supplementary material for: NLRX1 is a key regulator of immune signaling during invasive pulmonary aspergillosis
Source: PLoS Pathog. 2020 Sep 21;16(9):e1008854. doi: 10.1371/journal.ppat.1008854 (PMC7529209; doi:10.1371/journal.ppat.1008854)

## A CD45+ Gating Strategy

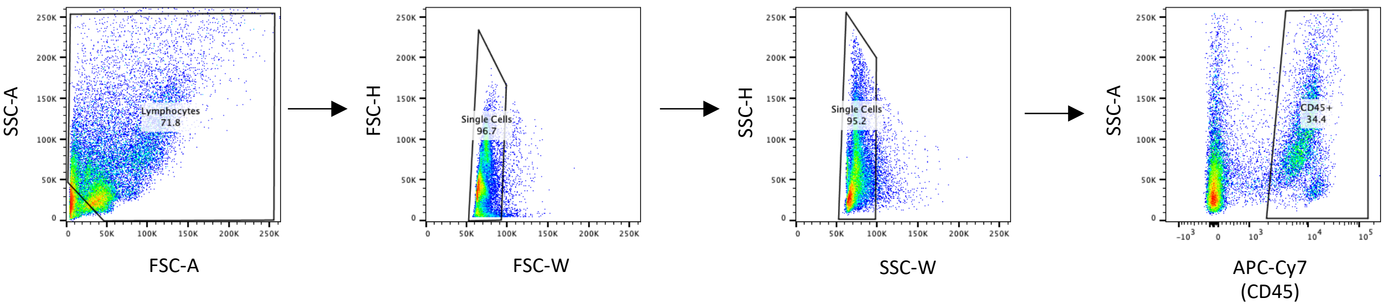


## B Panel 1 Gating Strategy

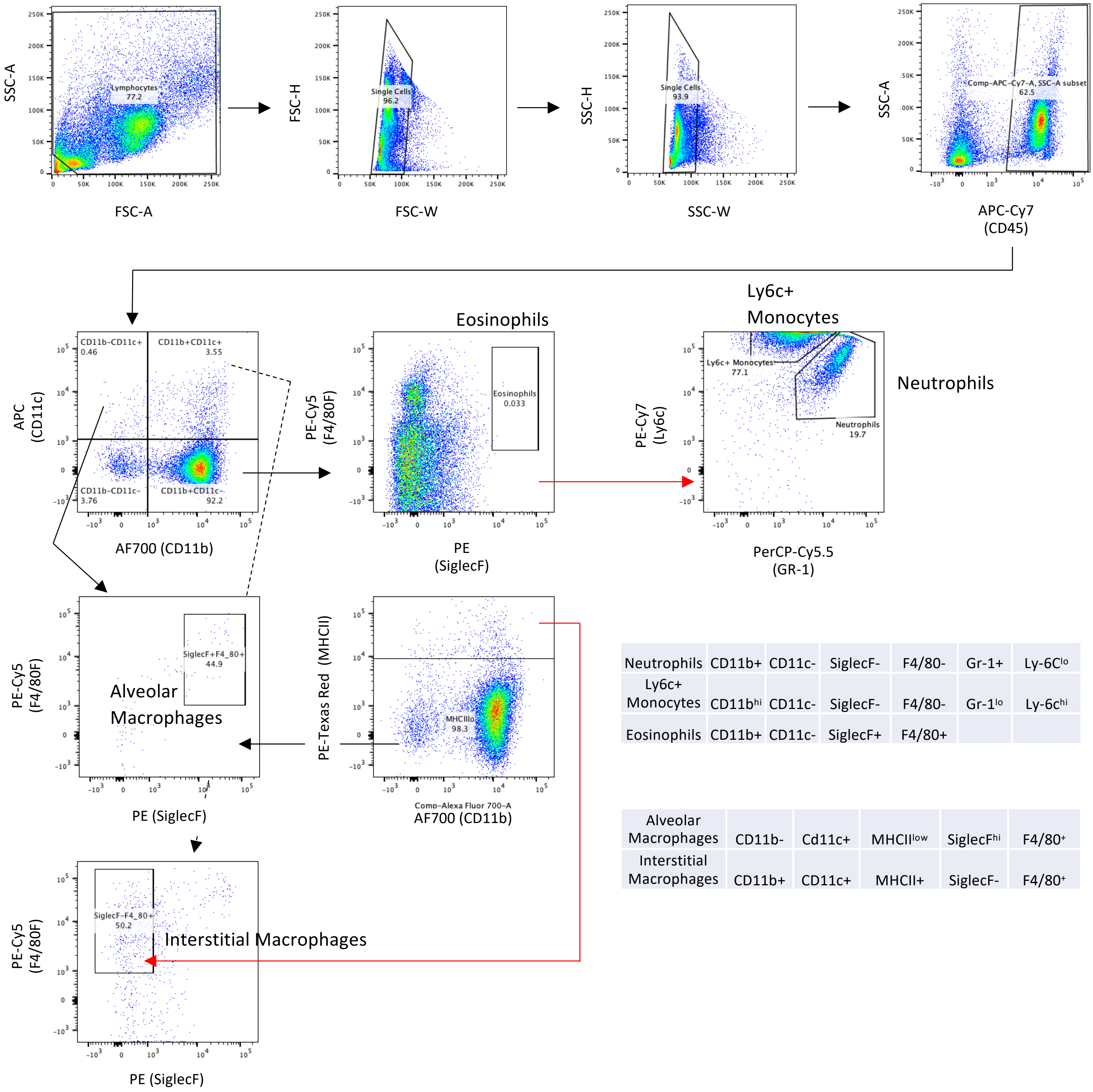


| | | | | | | |
|---|---|---|---|---|---|---|
| Neutrophils | CD11b+ | CD11c- | SiglecF- | F4/80- | Gr-1+ | Ly-6C$^{lo}$ |
| Ly6c+ Monocytes | CD11b$^{hi}$ | CD11c- | SiglecF- | F4/80- | Gr-1$^{lo}$ | Ly-6c$^{hi}$ |
| Eosinophils | CD11b+ | CD11c- | SiglecF+ | F4/80+ | | |

| | | | | | |
|---|---|---|---|---|---|
| Alveolar Macrophages | CD11b- | Cd11c+ | MHCII$^{low}$ | SiglecF$^{hi}$ | F4/80$^{+}$ |
| Interstitial Macrophages | CD11b+ | CD11c+ | MHCII+ | SiglecF- | F4/80$^{+}$ |

# C Panel 2 Gating Strategy

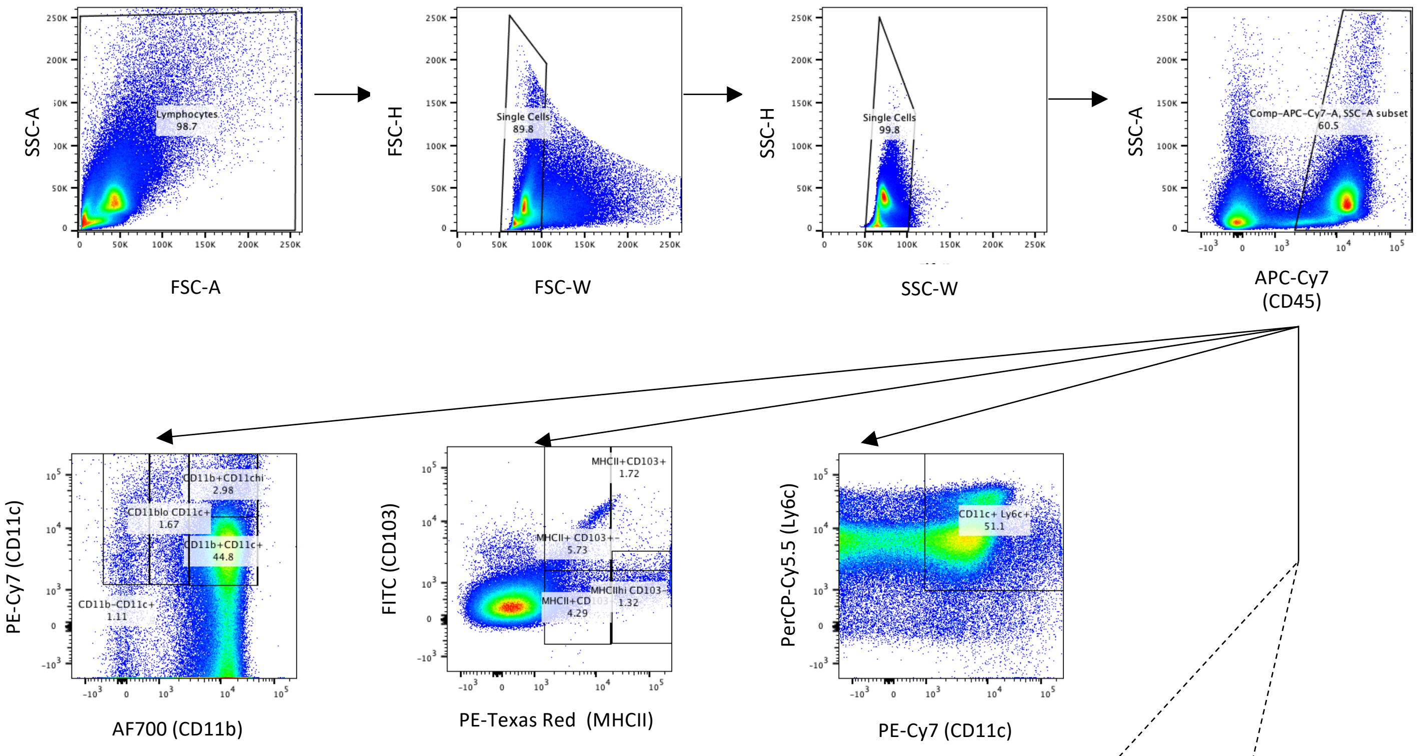


| | | | | | |
|---|---|---|---|---|---|
| pDC | CD11blo | CD11c+ | MHCII+ | Ly6c+ | |
| mDC | CD11b+ | CD11c+ | MHCIIhi | Ly6c+ | CD103- |
| cDC | CD11b+ | CD11chi | MHCII+ | | CD103- |
| CD103+ | CD11b- | CD11c+ | MHCII+ | | CD103+ |

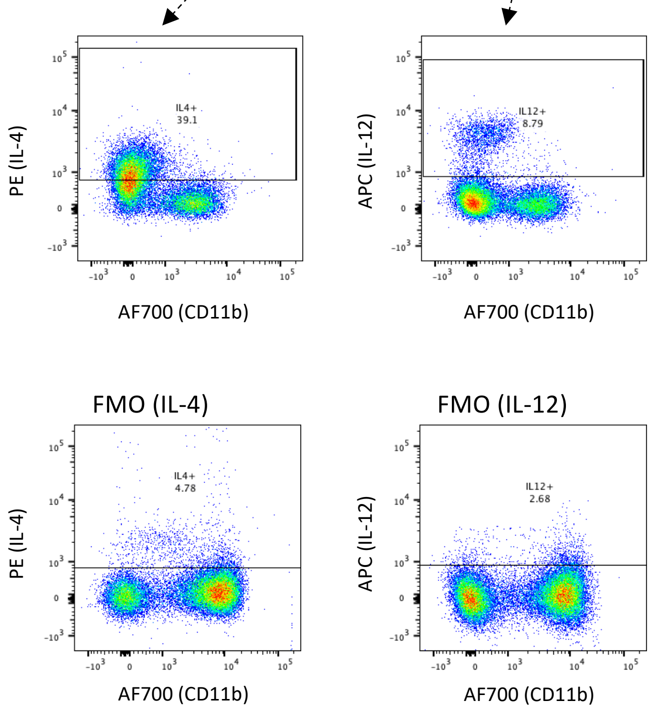

**D** **Panel 3 Gating Strategy**

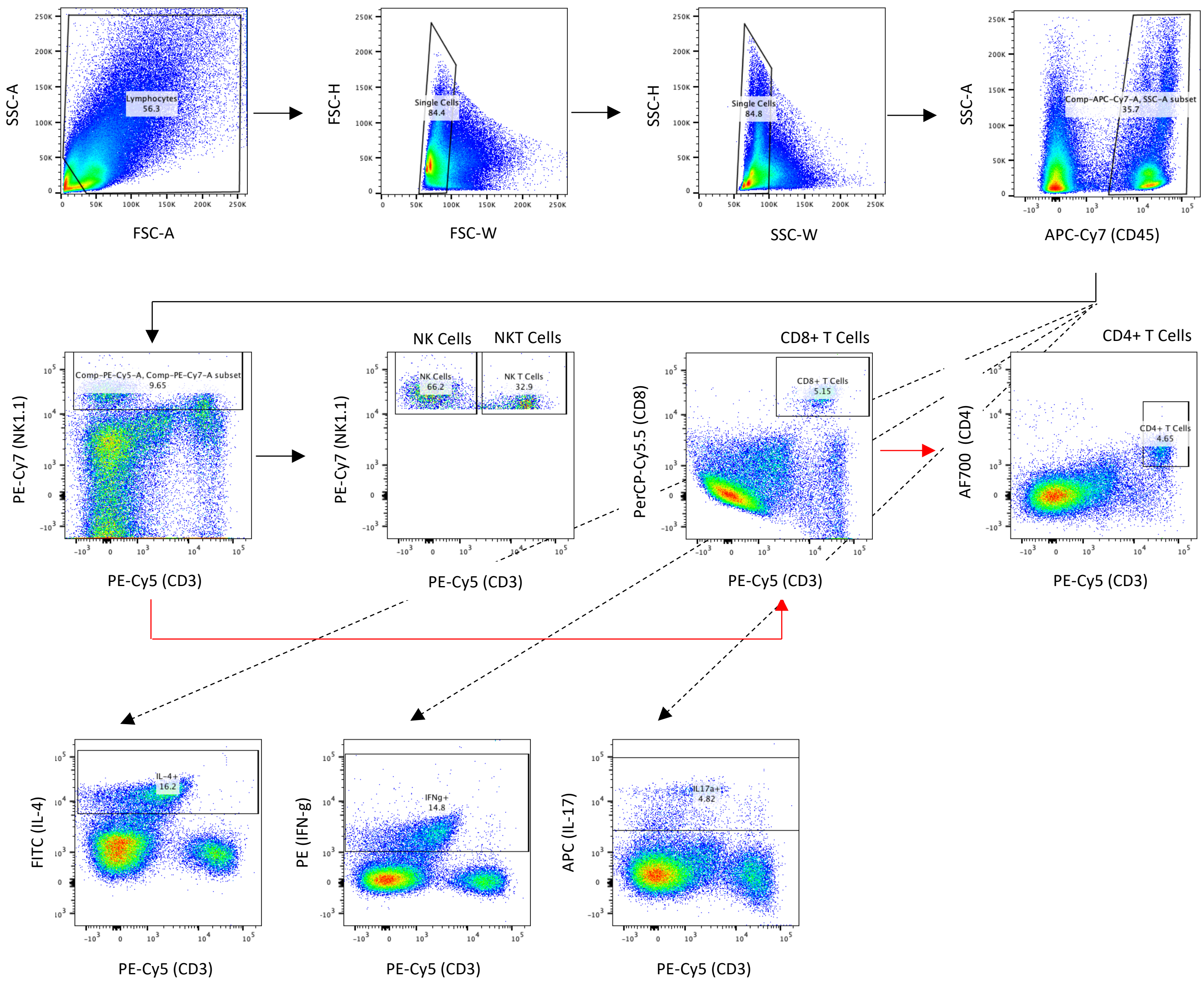

Supplement: S2 Fig — Four antibody panels were utilized to determine the identity of leukocyte populations. (A) Panel outline to determine percentage of CD45+ cells. (B) Gating strategy to identify alveolar macrophages, interstitial macrophages, neutrophils, eosinophils, and Ly6C+ monocytes. (C) Gating strategy to identify CD103+ dendritic cells, plasmocytoid dendritic cells, monocytoid dendritic cells, and conventional dendritic cells. Intra-cellular production of IL-4 and IL-12 is also determined for each cell population using Fluorescence minus one (FMO). (D) Gating strategy to identify natural killer cells, natural killer T cells, CD8+ T cells, and CD4+ T cells. Intra-cellular production of IL-4, IL-17a, and IFN-γ is also determined for each cell population. (PDF) [file ppat.1008854.s002.pdf]
